# Supplementary material for: One-shot preparation of topologically chimeric nanofibers via a gradient supramolecular copolymerization
Source: Nat Commun. 2019 Oct 8;10:4578. doi: 10.1038/s41467-019-12654-z (PMC6783438; doi:10.1038/s41467-019-12654-z)
Supplement: Supplementary file 1 — Supplementary Information [file 41467_2019_12654_MOESM1_ESM.pdf]

Supplementary Information

One-shot preparation of topologically chimeric nanofibers via a gradient supramolecular copolymerization

Yuichi Kitamoto[1], Ziyan Pan[2], Deepak D. Prabhu[2], Atsushi Isobe[2], Tomonori Ohba[3], Nobutaka Shimizu[4], Hideaki Takagi[4], Rie Haruki[4], Shin-ichi Adachi[4] and Shiki Yagai[1,2]*

[1] *Institute for Global Prominent Research (IGPR), Chiba University, 1-33, Yayoi-cho, Inage-ku, Chiba 263-8522, Japan.*
[2] *Division of Advanced Science and Engineering, Graduate School of Engineering, Chiba University, 1-33 Yayoi-cho, Inage-ku, Chiba 263-8522, Japan.*
[3] *Department of Chemistry, Graduate School of Science, Chiba University, 1-33 Yayoi-cho, Inage-ku, Chiba 263-8522, Japan.*
[4] *Photon Factory, Institute of Materials Structure Science, High Energy Accelerator Research Organization, Tsukuba 305-0801, Japan.*

**E-mail: yagai@faculty.chiba-u.jp*

This PDF file includes:

Supplementary Methods

Synthesis

Supplementary Figures and Tables

Supplementary References

Supplementary Methods

General methods.

^1H and ^{13}C NMR spectra were recorded on JEOL JNM-ECA500 NMR spectrometers at 500 and 125 MHz, respectively. ^1H NMR chemical shifts reported in ppm (δ) were referenced to the chemical shift of tetramethylsilane at 0.00 ppm. The resonance multiplicity is described as s (singlet), d (doublet), m (multiplet), br (broad), and brs (broad singlet). ^{13}C NMR chemical shifts reported in ppm (δ) were referenced to the chemical shifts of $CDCl_3$ at 77.16 ppm and THF-d_8 at 67.21 ppm. ESI-HRMS spectra were measured on an Exactive (Thermo Scientific).

UV–Vis spectroscopy.

UV–Vis absorption spectra were recorded on a JASCO V660 spectrophotometer equipped with a JASCO ETCS-761 temperature controller using a screw capped quartz cuvette with optical path length of 1.0 cm.

Fluorescence spectroscopy.

Fluorescence spectra were measured with JASCO FP-8300 with a JASCO ETC-815 temperature controller using a screw capped quartz cuvette with optical path length of 1.0 cm.

Transmission electron microscopy (TEM).

TEM imaging was performed using a JEM-2100F (JEOL) at an acceleration voltage of 120 kV. TEM samples were prepared by spin-coating MCH solutions of supramolecular polymers onto elastic carbon-coated copper grid (Okenshoji Co. Ltd., SHR-C075 STEM Cu 75P, grade: super ultrahigh resolution carbon, mesh 339, whole size 75 μm) and dried under air for 1 h followed by drying under vacuum for 24 h.

Small Angle X-Ray Scattering (SAXS).

SAXS measurements were performed at BL-10C at the Photon Factory of the High Energy Accelerator Research Organization (KEK) in Tsukuba, Japan[1]. The MCH solutions of supramolecular polymers loaded into cells (stainless-steel surround, 20 μm-thick quartz glass windows, 1.25 mm-path length). The measurements were employed at around 293 K. An X-ray wavelength was adjusted to 1.5 Å and a sample-detector distance was 1029 mm. A calibration was performed using a silver behenate as a standard sample. These settings provided a detectable Q-range of order 0.1–5.9 nm^{-1}. Sixty frames were collected with the exposure time of 10 s. Radiation damages were not observed, and accordingly all the data were averaged to afford a total integration time of 600 s. The two-dimensional scattering data obtained by using a DECTRIS PILATUS3 2M detector were radially averaged to give one-dimensional scattering intensity data [$I(Q)$ vs. Q]. The normalization using water as a reference and the background (solvent + cell) subtraction gave absolute scattering intensity $I(Q)$ in cm^{-1}. All data reduction was performed using the *SAngler* software package[2].

Estimation of degree of polymerization (*DP*) of supramolecular polymers from AFM images. *DP* was estimated from AFM images of supramolecular polymer fibers and the optimized structure of rosettes by force-filed molecular mechanics calculations. The following figure is a schematic representation of stacked rosettes to form supramolecular fiber[3].

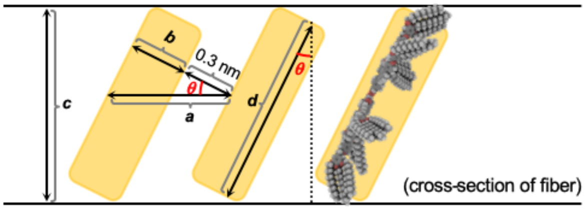

The calculation of *DP* per rosette (DP_{rosette}) is first estimated as follows. The distance between rosettes along the fiber axis (a) is given as:

$$a = \frac{b + 0.3}{cos\theta} \quad (1)$$

In this Supplementary Equation, b is the thickness of molecular modeled rosettes and 0.3 is a possible distance (nm) of π–π stacking. Based on the height (thickness) of fibers measured by AFM (c), $cos\ \theta$ is given as:

$$cos\theta = \frac{c}{d} \quad (2)$$

Here, d is the diameter of molecular modeled rosettes. Accordingly, Supplementary Equation 1 can be expressed as:

$$a = \frac{d(b + 0.3)}{c} \quad (3)$$

The average length of fibers (L_{av}) can be calculated as follows:

$$L_{av} = a \times DP_{rosette} \quad (4)$$

The L_{av} values can be directly measured by analyzing AFM images. Therefore, from Supplementary Equations 3,4, *DP* per monomer ($DP_{\text{mon}} = 6DP_{\text{rosette}}$) can be calculated according to Supplementary Equation 5:

$$DP_{mon} = \frac{6L_{av}}{a} = \frac{6cL_{av}}{d(b + 0.3)} \quad (5)$$

Synthesis

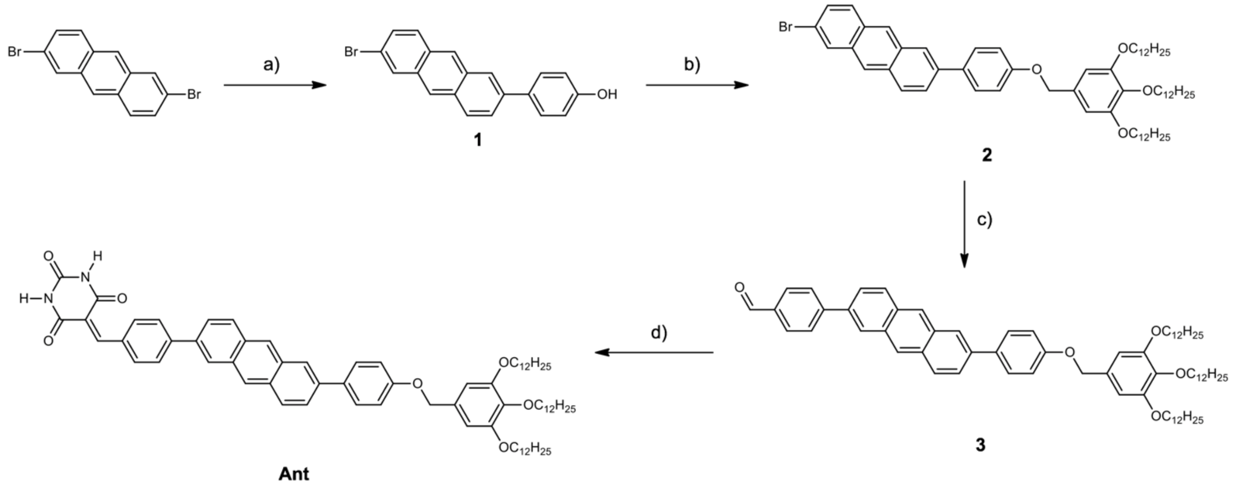

Reagents and conditions: a) 4-(4,4,5,5-tetramethyl-1,3,2-dioxaborolan-2-yl)phenol, $Pd(PPh_3)_4$, tri(*p*-tolyl)phosphine, Na_2CO_3, THF–H_2O, 70 °C, 15 h; b) 5-(chloromethyl)-1,2,3-tris-(dodecyloxy)benzene[4], K_2CO_3, DMF, 75 °C, 12 h; c) 4-formylphenylboronic acid, $Pd_2(dba)_3{\cdot}CHCl_3$, SPhos (2-dicyclohexylphosphino-2′,6′-dimethoxybiphenyl), Na_2CO_3, THF–H_2O, 70 °C, 3 h; d) barbituric acid, ethanol, reflux, 20 h.

4-(6-bromoanthracen-2-yl)phenol (1): A 50 mL three neck round bottom flask connected with Dimroth condenser was charged with 2,6-dibromoanthracene (300 mg, 8.93×10^{-1} mmol), 4-(4,4,5,5-tetramethyl-1,3,2-dioxaborolan-2-yl)phenol (111 mg, 8.03×10^{-1} mmol, 0.9 equiv.), $Pd(PPh_3)_4$ (52 mg, 4.46×10^{-2} mmol, 5 mol%), tri(*p*-tolyl)phosphine (14 mg, 4.46×10^{-2} mmol, 5 mol%), and Na_2CO_3 (284 mg, 2.68 mmol, 3.0 equiv.). The flask was evacuated and refilled three times with N_2. THF–H_2O (4:1 mixture, 20 mL) was added, and then stirred at 70 °C for 15 h. After the reaction mixture was diluted with ethyl acetate, the resulting solution was washed with sat. NH_4Cl aq., and brine, dried over Na_2SO_4, and evaporated. The residue was purified by silica gel chromatography using *n*-hexane–acetone (4:1) as an eluent to give the product **1** (144 mg, 51%) as yellow solids.

^{1}H NMR (500 MHz, THF-d_8, 293 K): δ (ppm) 8.47 (s, 1H), 8.43 (brs, 1H), 8.34 (s, 1H), 8.22 (d, 1H, J = 1.2 Hz), 8.17 (d, 1H, J = 0.9 Hz), 8.05 (d, 1H, J = 9.0 Hz), 7.93 (d, 1H, J = 9.0 Hz), 7.79 (dd, 1H, J = 8.8, 1.8 Hz), 7.64 (d, 2H, J = 8.7 Hz), 7.49 (dd, 1H, J = 8.8, 1.9 Hz), 6.87 (d, 2H, J = 8.7 Hz); ^{13}C NMR (125 MHz, THF-d_8, 293 K): δ (ppm) 158.59, 138.99, 133.28, 133.04, 132.25, 132.04, 131.00, 130.65, 130.54, 129.25, 129.12, 128.76, 127.12, 126.70, 125.73, 124.60, 119.54, 116.41; ESI-HRMS (m/z): calcd. for $C_{20}H_{14}BrO$ $[M+H]^+$: 349.0223, found 349.0069.

2-bromo-6-(4-((3,4,5-tris(dodecyloxy)benzyl)oxy)phenyl)anthracene (2): A 50 mL three neck round bottom flask was charged with compound **1** (119 mg, 3.40×10^{-1} mmol, 1.1 equiv.) and K_2CO_3 (214 mg, 1.55 mmol, 5.0 equiv.). The flask was evacuated and refilled three times with N_2. Dry DMF (8 mL) was added to the flask, and the mixture was stirred at 75 °C for 1 h. After the

addition of 5-(chloromethyl)-1,2,3-tris-(dodecyloxy)benzene[4] (210 mg, 3.09 × 10^{-1} mmol), the mixture was further stirred at 75 °C for 12 h. After H_2O was added to the reaction mixture, the aqueous phase was washed with ethyl acetate–*n*-hexane (1:4) four times. The combined organic phase was dried over Na_2SO_4, and evaporated. The residue was purified by silica gel chromatography using *n*-hexane–acetone (4:1) as an eluent to give the product **2** (284 mg, 93%) as yellow solids.

^{1}H NMR (500 MHz, $CDCl_3$, 293 K): δ (ppm) 8.40 (s, 1H), 8.31 (s, 1H), 8.16 (s, 1H), 8.12 (s, 1H), 8.04 (d, 1H, J = 8.9 Hz), 7.86 (d, 1H, J = 9.0 Hz), 7.75 (dd, 1H, J = 8.8, 1.5 Hz), 7.70 (d, 2H, J = 8.8 Hz), 7.50 (dd, 1H, J = 8.8, 1.7 Hz), 7.11 (d, 2H, J = 8.7 Hz), 6.66 (s, 2H), 5.02 (s, 2H), 4.01–3.95 (m, 6H), 1.83–1.72 (m, 6H), 1.50–1.26 (m, 54H), 0.89–0.86 (m, 9H); ^{13}C NMR (125 MHz, $CDCl_3$, 293 K): δ (ppm) 158.78, 153.47, 138.14, 137.89, 133.64, 132.40, 132.24, 131.87, 130.30, 130.02, 129.94, 129.10, 128.77, 128.49, 126.74, 126.26, 125.26, 124.85, 119.45, 115.49, 106.30, 73.60, 70.73, 69.30, 32.10, 30.52, 29.94, 29.92, 29.88, 29.83, 29.60, 29.58, 29.55, 29.32, 26.28, 22.87, 14.30; ESI-HRMS (m/z): calcd. for $C_{63}H_{92}BrO_4$ $[M+H]^+$: 991.6174, found 991.6202.

4-(6-(4-((3,4,5-tris(dodecyloxy)benzyl)oxy)phenyl)anthracen-2-yl)benzaldehyde (3): A 50 mL three neck round bottom flask connected with Dimroth condenser was charged with compound **2** (170 mg, 1.71 × 10^{-1} mmol), 4-formylphenylboronic acid (31 mg, 2.60 × 10^{-1} mmol, 1.2 equiv.), $Pd_2(dba)_3{\cdot}CHCl_3$ (5 mg, 5.14 × 10^{-3} mmol, 3 mol%), SPhos (4 mg, 1.03 × 10^{-2} mmol, 6 mol%), and Na_2CO_3 (54 mg, 5.14 × 10^{-1} mmol mmol, 3.0 equiv.). The flask was evacuated and refilled three times with N_2. THF–H_2O (4:1 mixture, 7 mL) was added, and the mixture was stirred at 70 °C for 3 h. After the reaction mixture was diluted with chloroform, the resulting solution was washed with sat. NH_4Cl aq., and brine. The organic layer was dried over Na_2SO_4, and evaporated. The residue was purified by silica gel chromatography using *n*-hexane–acetone (1:1) as an eluent to give the product **3** (159 mg, 91%) as yellow solids.

^{1}H NMR (500 MHz, $CDCl_3$, 293 K): δ (ppm) 10.09 (s, 1H), 8.49 (s, 1H), 8.47 (s, 1H), 8.27 (s, 1H), 8.16 (s, 1H), 8.11 (d, 1H, J = 8.7 Hz), 8.08 (d, 1H, J = 8.7 Hz), 8.02 (d, 2H, J = 8.4 Hz), 7.94 (d, 2H, J = 8.2 Hz), 7.77–7.75 (m, 2H), 7.72 (d, 1H, J = 8.6 Hz), 7.12 (d, 2H, J = 8.8 Hz), 6.66 (s, 2H), 5.03 (s, 2H), 4.01–3.95 (m, 6H), 1.83–1.73 (m, 6H), 1.50–1.26 (m, 54H), 0.90–0.86 (m, 9H); ^{13}C NMR (125 MHz, $CDCl_3$, 293 K): δ (ppm) 192.02, 158.78, 153.47, 147.13, 138.13, 137.91, 136.25, 135.34, 133.69, 132.63, 131.88, 131.65, 131.56, 131.30, 130.52, 129.26, 128.89, 128.51, 127.93, 127.01, 126.97, 126.35, 125.97, 125.02, 124.88, 115.50, 106.30, 73.60, 70.73, 69.30, 32.10, 30.52, 29.94, 29.92, 29.88, 29.84, 29.60, 29.57, 29.54, 26.32, 26.28, 22.87, 14.30; ESI-HRMS (m/z): calcd. for $C_{70}H_{97}O_5$ $[M+H]^+$: 1017.7331, found 1017.7344.

5-(4-(6-(4-((3,4,5-tris(dodecyloxy)benzyl)oxy)phenyl)anthracen-2-yl)benzylidene)pyrimidine-2,4,6(1H,3H,5H)-trione (Ant): A 50 mL three neck round bottom flask connected with Dimroth condenser was charged with compound **3** (88 mg, 8.85 × 10^{-2} mmol) and barbituric acid (56 mg, 4.42 × 10^{-1} mmol, 5.0 equiv.). Ethanol (25 mL) was added, and then refluxed for 20 h. After the

reaction mixture was cooled to room temperature, the resulting precipitate was filtered, and washed several times with hot ethanol. The residue was dissolved with chloroform. Acetone–*n*-hexane (1:2) was added, and then reprecipitated at 0 °C. The resulting precipitate was collected by centrifuge and washed with acetone–*n*-hexane (1:2). This purification protocol was repeated at least three times to give **Ant** (48 mg, 49 %) as orange solids.

^{1}H NMR (500 MHz, $CDCl_3$, 333 K): δ (ppm) 8.63 (s, 1H), 8.48 (s, 1H), 8.44 (s, 1H), 8.37 (d, 2H, *J* = 8.4 Hz), 8.30 (s, 1H), 8.14 (s, 1H), 8.08 (d, 1H, *J* = 8.9 Hz), 8.06 (d, 1H, *J* = 8.8 Hz), 7.91–7.89 (m, 3H, (brs signal of N-*H* overlapped)), 7.78–7.74 (m, 3H (brs signal of N-*H* overlapped)), 7.70 (d, 2H, *J* = 8.6 Hz), 7.10 (d, 2H, *J* = 8.7 Hz), 6.65 (s, 2H), 5.03 (s, 2H), 4.01–3.96 (m, 6H), 1.82–1.72 (m, 6H), 1.50–1.45 (m, 6H), 1.38–1.27 (m, 48H), 0.90–0.86 (m, 9H); ^{13}C NMR (125 MHz, $CDCl_3$, 333 K): δ (ppm) 159.93, 153.68, 135.90, 135.86, 133.93, 132.96, 132.09, 131.86, 131.54, 129.38, 128.94, 128.57, 127.32, 127.22, 127.17, 126.42, 126.09, 124.98, 124.80, 115.83, 106.97, 73.71, 70.94, 69.75, 32.12, 30.63, 29.94, 29.92, 29.89, 29.84, 29.78, 29.65, 29.56, 29.53, 26.39, 26.36, 22.85, 14.18; ESI-HRMS (m/z): calcd. for $C_{74}H_{102}N_3O_7$ $[M+NH_4]^+$: 1144.7712, found 1144.7722.

Supplementary Figures and Tables

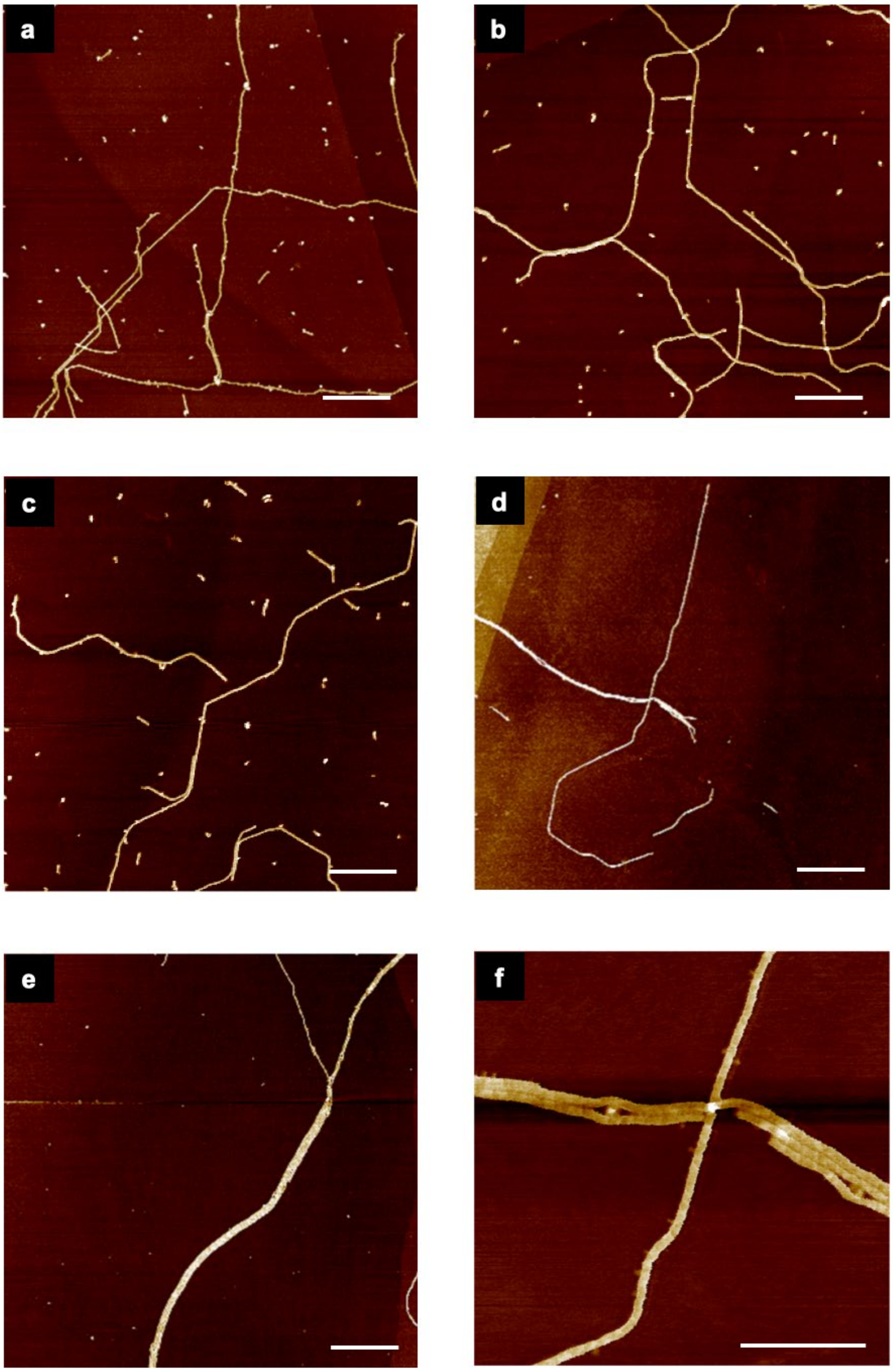

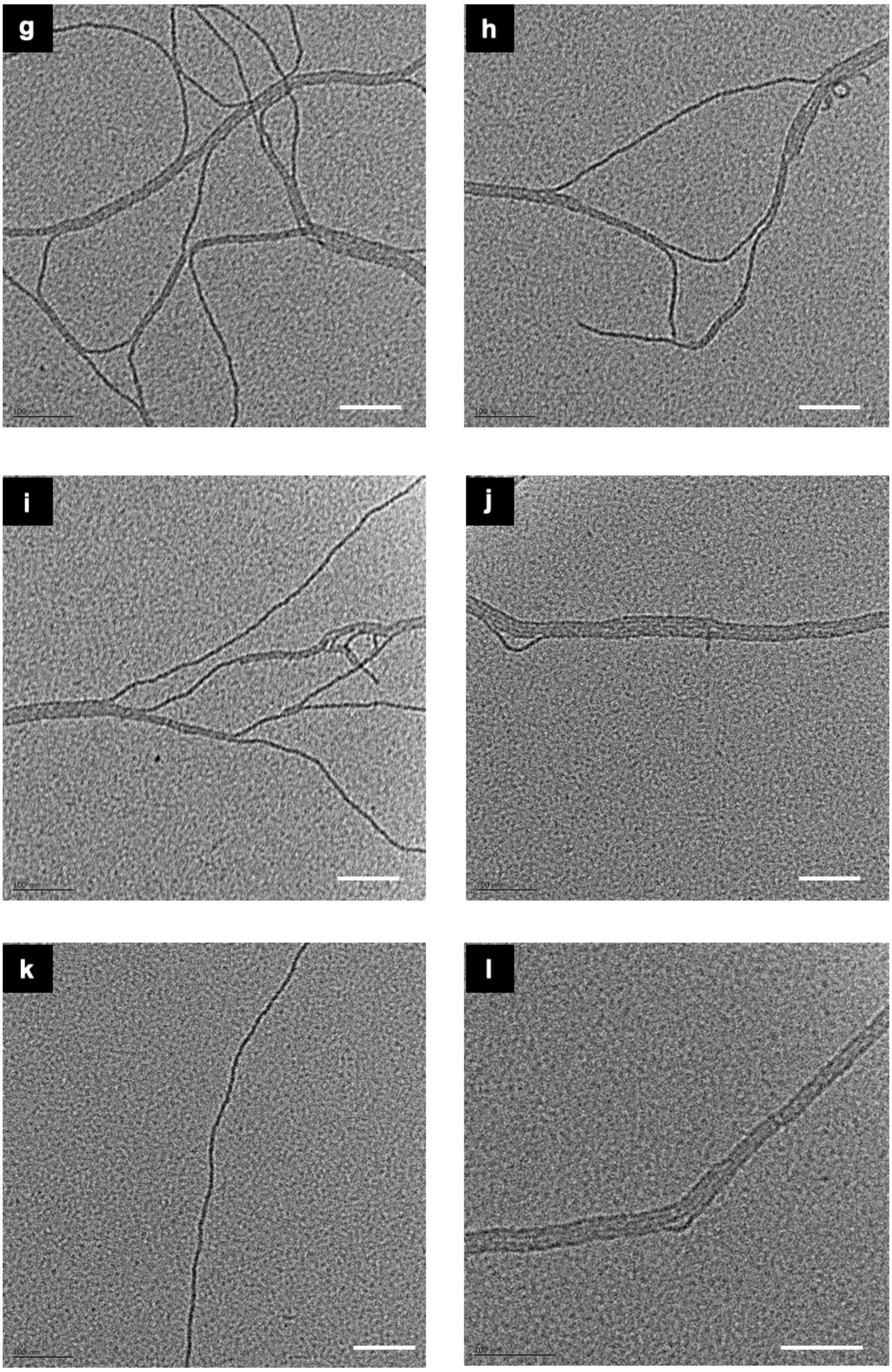

Supplementary Figure 1. Additional AFM and TEM images of the supramolecular polymers of Ant. a–f Additional AFM images of supramolecular polymers of **Ant**, which were prepared by cooling a hot MCH solution of **Ant** ($c = 1.0 \times 10^{-5}$ M) from 373 to 293 K at a cooling rate of 1.0 K min^{-1}. The sample was prepared by spin-coating the solution onto highly oriented pyrolytic graphite (HOPG). Scale bars, 300 nm (**a**–**e**) or 100 nm (**f**). **g**–**l** TEM images of supramolecular polymers of **Ant**, which were prepared by the same procedure to the AFM measurements. Scale bars, 100 nm.

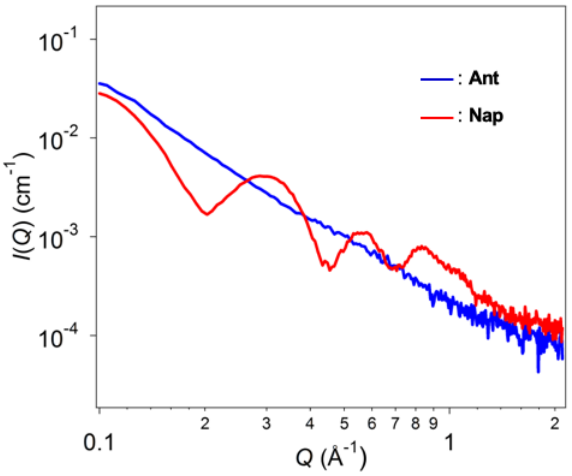

Supplementary Figure 2. SAXS profiles of supramolecular homopolymers. SAXS profiles of MCH solutions of **Nap** (red line) and **Ant** (blue line) (c = 5.0 × 10^{-5} M) prepared by cooling from 373 to 293 K at a cooling rate of 1.0 K min^{-1}.

a

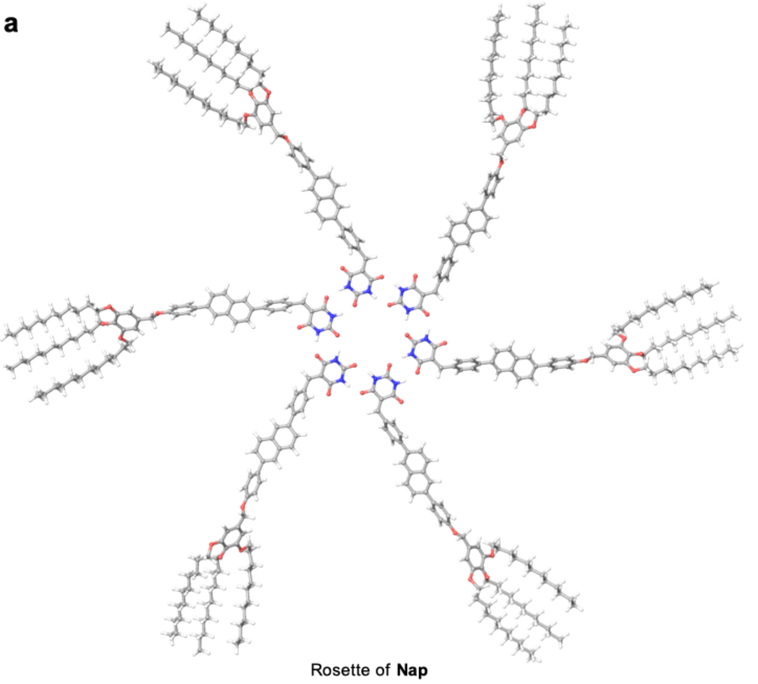

Rosette of **Nap**

c

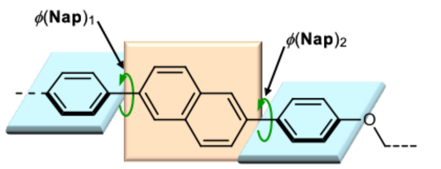

	ϕ(**Nap**)1	ϕ(**Nap**)2
twisting angle (°)	46.6–47.1	47.9–48.5

b

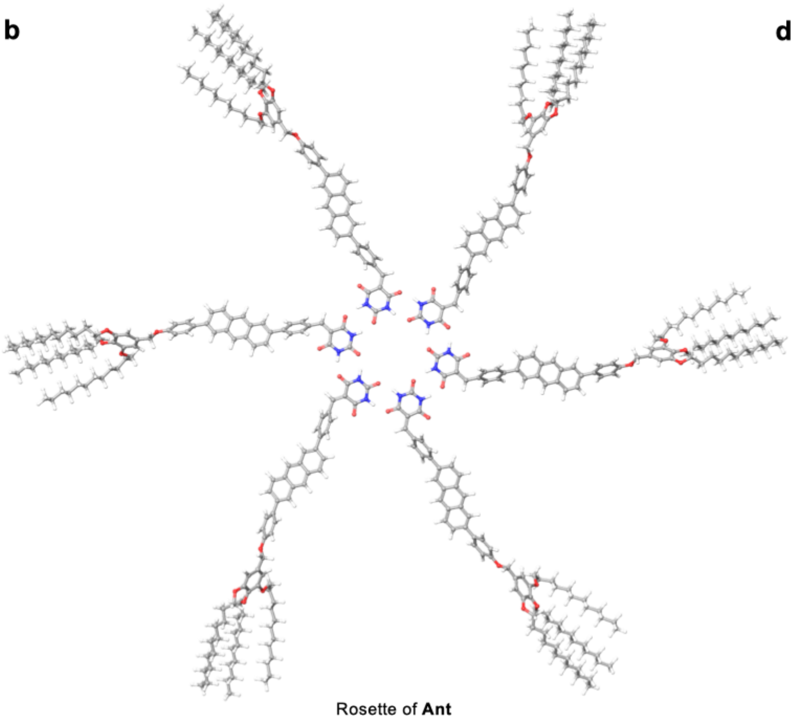

Rosette of **Ant**

d

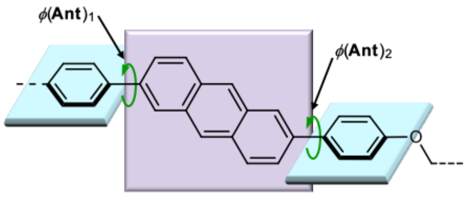

	ϕ(**Ant**)1	ϕ(**Ant**)2
twisting angle (°)	48.2–48.4	47.0–47.3

Supplementary Figure 3. Geometry-optimized structure of rosettes. a,**b** Geometry-optimized structures of rosettes of **Nap** (**a**) and **Ant** (**b**) by AMBER force-field calculations. **c**,**d** The twisting angles of the phenyl–acene–phenyl moieties in the geometry optimized rosettes of **Nap** (**c**) and **Ant** (**d**). The hydrogen-bonded arrangement of the rosettes are based on those visualized by STM for related compounds lacking long aliphatic chains[5,6].

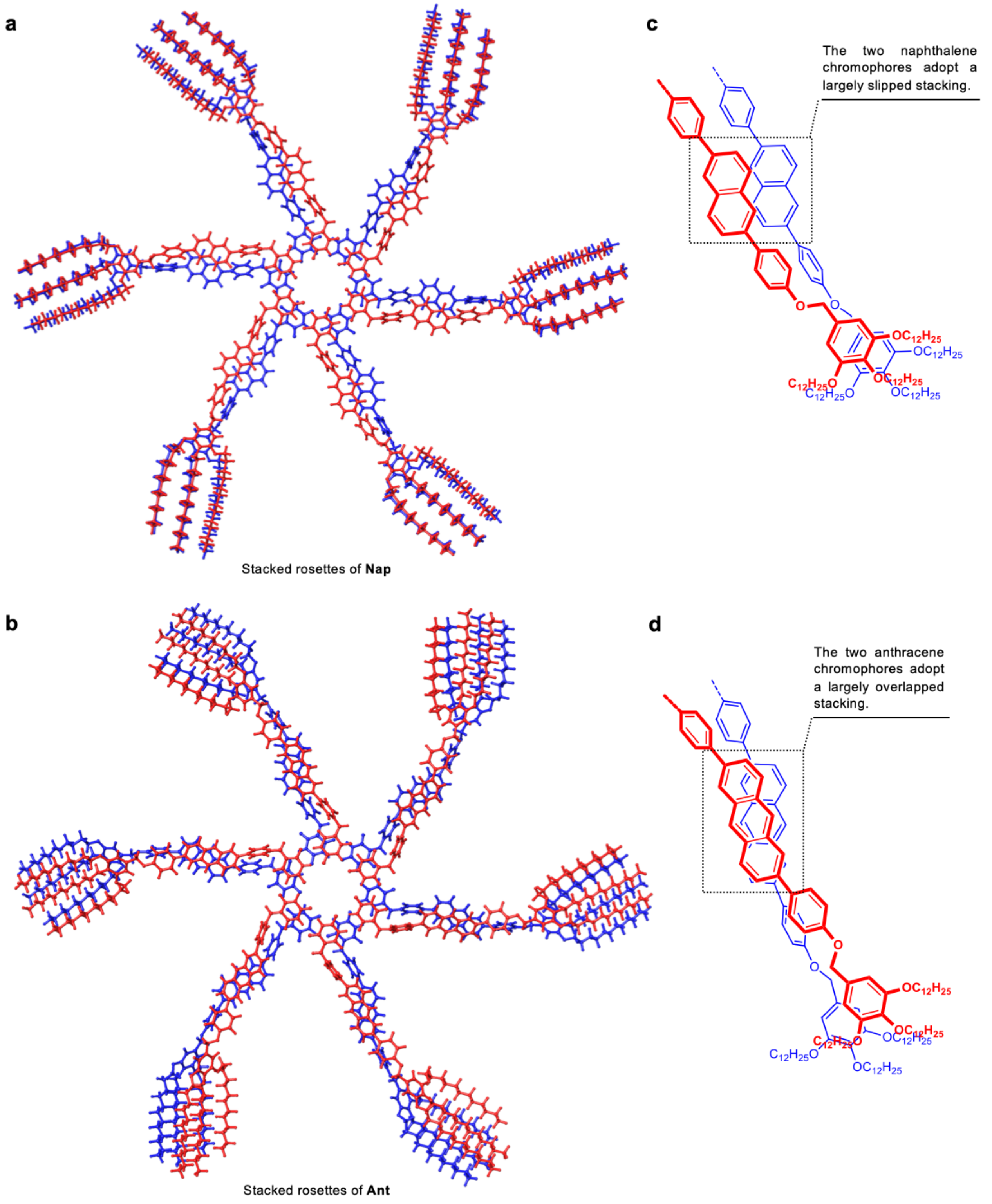

Supplementary Figure 4. Geometry-optimized stacks of two rosettes. a,**b** Geometry-optimized stacking structures of two rosettes of **Nap** (**a**) and **Ant** (**b**) by AMBER force-field calculations. Upper and lower rosettes are colored by red and blue, respectively. **c**,**d** Schematic representation of the stacking arrangement of acene moieties in (**c**) and (**d**), respectively.

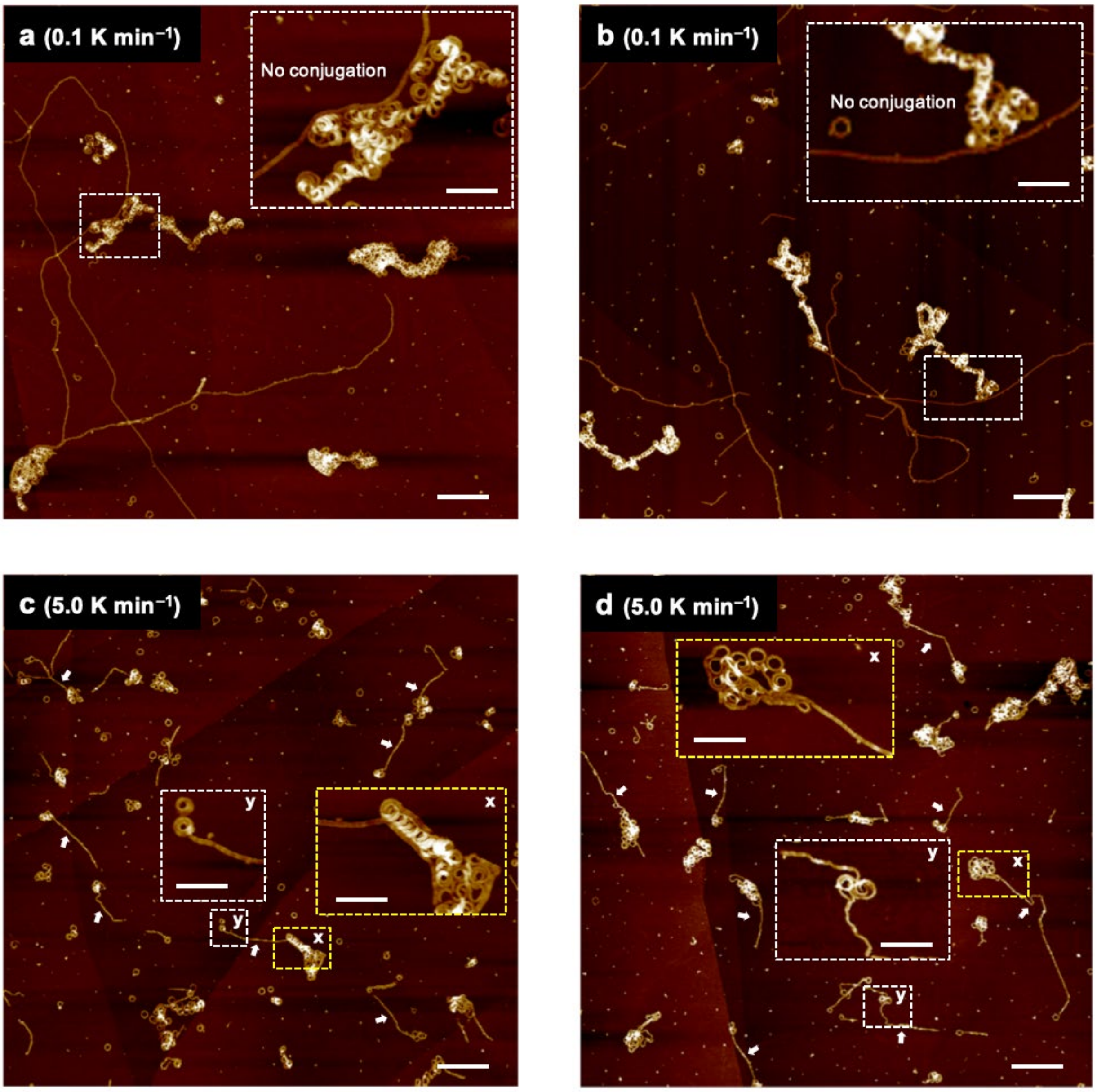

Supplementary Figure 5. Cooling-rate-dependence of supramolecular copolymerization in the 1:1.5 mixture of Ant and Nap. AFM images of supramolecular (co)polymers prepared by cooling a hot MCH solution of 1:1.5 mixture of **Ant** and **Nap** ([**Ant**] = 1.0 × 10^{-5} M; [**Nap**] = 1.5 × 10^{-5} M) from 373 to 293 K at cooling rates of 0.1 K min^{-1} (**a**,**b**), and 5.0 K min^{-1} (**c**,**d**). The samples were prepared by spin-coating the solutions onto HOPG. The inset images show magnification of the area enclosed by the dashed rectangles. Scale bars, 300 nm (100 nm for inset images).

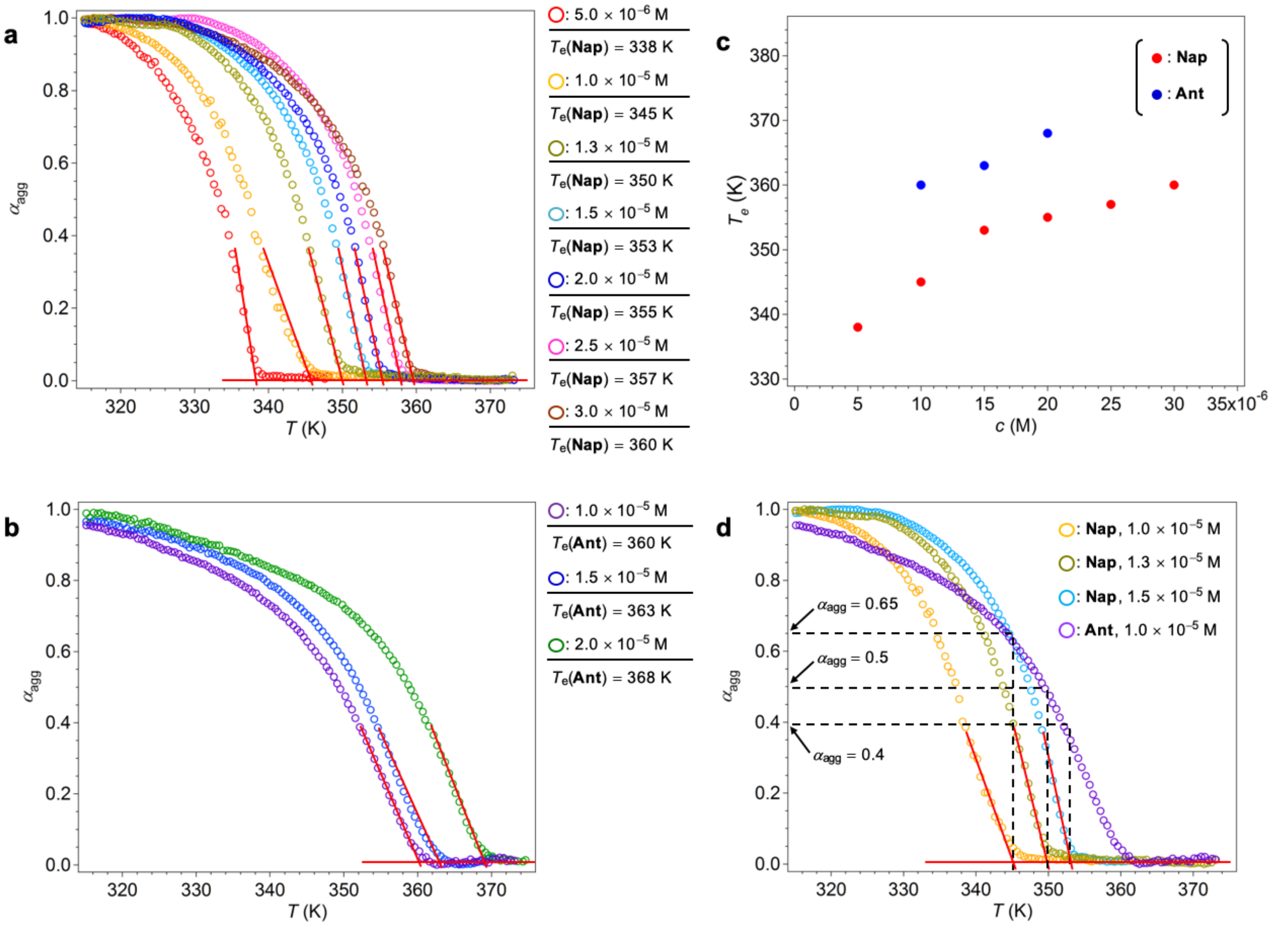

Supplementary Figure 6. Supramolecular polymerization process studied by UV-Vis spectroscopy. **a**,**b** Cooling curves of **Nap** (**a**) and **Ant** (**b**) obtained by plotting the mole fraction of the aggregated molecules (α_{agg}, calculated from the absorption change at λ = 470 nm) as a function of the temperature at different concentrations. The cooling rate was 1.0 K min^{-1}. Upon heating the as-prepared supramolecular polymer solution by the heating rate of 1.0 K min^{-1}, a remarkable thermal hysteresis was observed[7]. For such cooling processes that are under kinetic control, elongation temperature (T_e) cannot be estimated using a cooperative model fitting assuming the thermodynamic equilibrium[8]. Because sharp increase of α_{agg} is characteristic for a cooperative supramolecular polymerization and T_e is defined as a critical point that separates the nucleation and elongation processes, we manually estimated the T_e values from intersection temperature between a baseline of α_{agg} = 0 and a tangent line where $|\Delta\alpha_{agg}/\Delta T|$ gets maximum. **c** Concentration-dependence of T_e of **Nap** (red dots) and **Ant** (blue dots). **d** Overlay of the cooling curve of **Ant** (c = 1.0 × 10^{-5} M) and those of **Nap** at various concentrations to show how α_{agg} of **Ant** at the T_e of **Nap** changes upon changing the concentration of **Nap**.

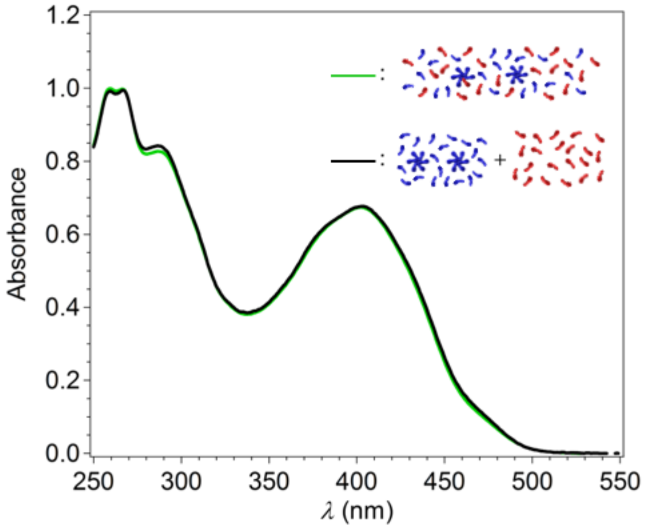

Supplementary Figure 7. Comparison of the UV-vis absorption spectra of pure Ant and a mixture with Nap. UV-vis absorption spectrum for a 1:1.5 mixture of **Ant** and **Nap** at 361 K (green curve, [**Ant**] = 1.0 × 10^{-5} M; [**Nap**] = 1.5 × 10^{-5} M) in MCH obtained by cooling the solution from 373 to 361 K using a cooling rate of 0.1 K min^{-1}. The black curve corresponds to a reference curve obtained by a simple summation of the absorption spectra of the two homoassemblies prepared by cooling each solution from 373 to 358 K using a cooling rate of 0.1 K min^{-1}.

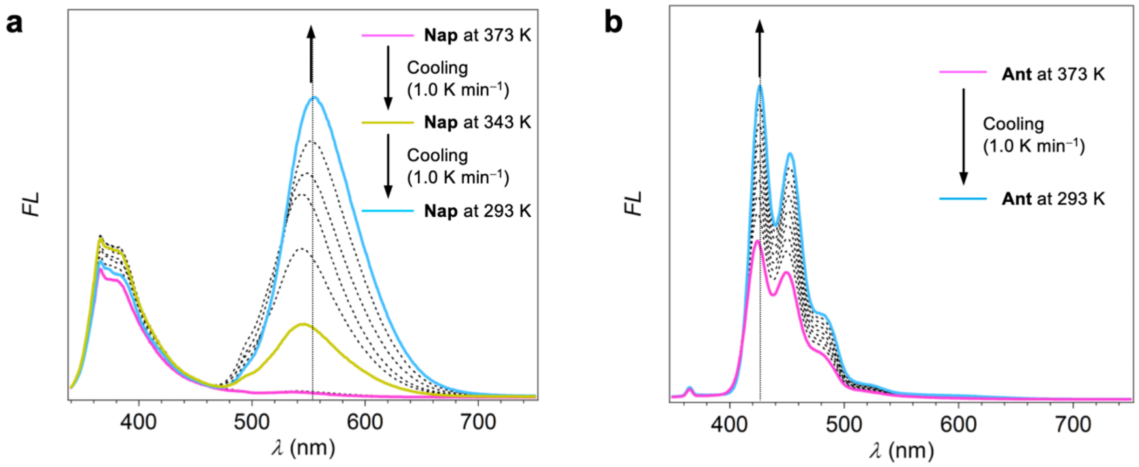

Supplementary Figure 8. Temperature-dependent fluorescence properties of Ant and Nap. Temperature-dependent fluorescence spectra of **Ant** (**a**) and **Nap** (**b**) (c = 1.0 × 10^{-5} M) upon cooling from 373 to 293 K at a cooling rate of 1.0 K min^{-1}. The excitation wavelength was 330 nm.

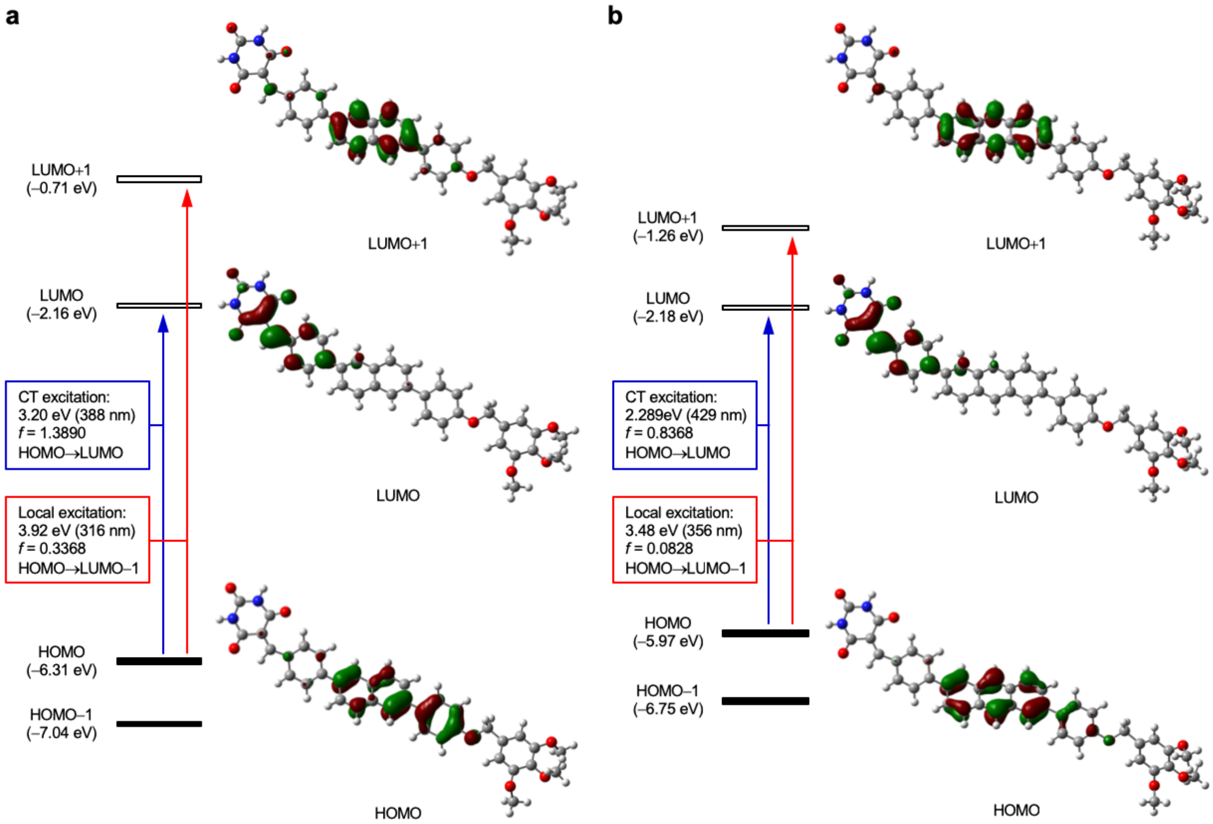

Supplementary Figure 9. Comparison of Kohn-Sham orbitals and energy levels. The molecular structures of **Nap** (**a**) and **Ant** (**b**) were optimized by DFT calculation using the Gaussian 16 program[9] at the BMK/6-311G(d,p) level of theory (Temperature = 373 K; in MCH, PCM model). The time-dependent DFT calculations of the two monomers were also performed at the same level of theory. For all calculations, the dodecyl groups were replaced with methyl groups. A number of functionals, including B3LYP (20% HF), CAM-B3LYP (11% HF (short), 65% HF (long)), PBE1PBE (25%), M06 (27% HF), BMK (42% HF), M06-2X (54% HF), were pretested, and all these functions afforded a similar tendency that the CT excitation (HOMO→LUMO) has a smaller excitation energy compared with the local excitation (see below). The excitation transition obtained by BMK showed the most satisfactory agreement with the experimental values of the monomeric state. Accordingly, we here showed the calculated results using BMK as a function.

For both monomers, the CT excitation (HOMO→LUMO) between the barbiturated phenyl moiety and the phenyl acene moiety (blue arrows) show a smaller excitation energy compared with the local excitation between the acene moiety and the phenyl acene moiety (red arrows). Accordingly, the longer wavelength absorption bands of **Nap** (> 380 nm, Fig. 2g) and **Ant** (> 420 nm, Fig. 2h) in the experimental absorption spectra of the monomer (at 373 K) can be attributed to the intramolecular CT transition (HOMO→LUMO). Because the FL spectra of the monomers (at 373 K, Supplementary Fig. 8) showed emission bands in shorter wavelength region than the CT absorption bands, the observed monomer emission can be attributed to the radiation from the locally excited state.

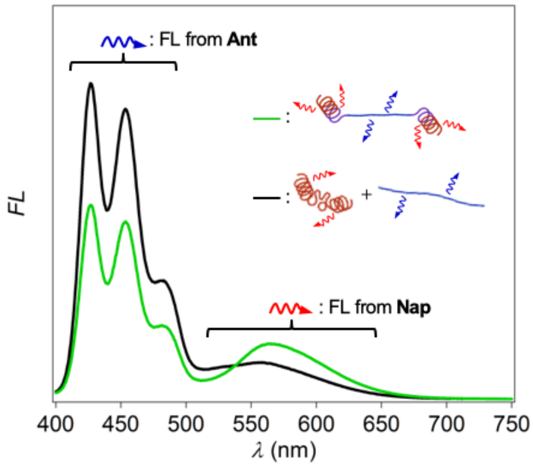

Supplementary Fig. 10. Comparison of fluorescence spectra of chimera fibers and a mixture of homopolymers. Fluorescence spectra of a 1:1.5 mixture of **Ant** and **Nap** ([**Ant**] = 1.0 × 10^{-5} M; [**Nap**] = 1.5 × 10^{-5} M) at 293 K. Green line: fluorescence spectrum of a mixture prepared by the gradient supramolecular copolymerization condition. Black line: fluorescence spectrum of a mixture prepared by mixing the separately prepared two homopolymer solutions. The excitation wavelength was 298 nm, at which **Ant** is predominantly excited.

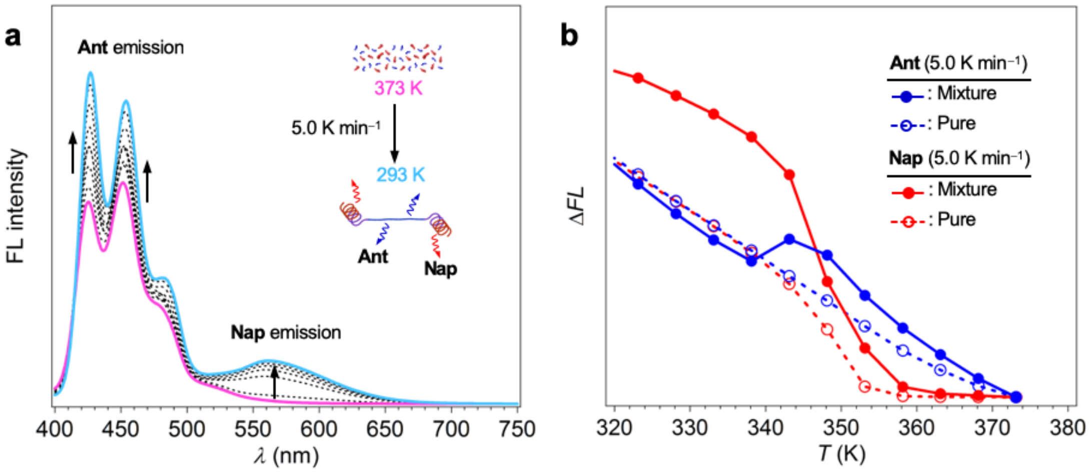

Supplementary Figure 11. Temperature-dependent fluorescence properties of 1:1.5 mixtures of Ant and Nap prepared by the cooling rate of 5.0 K min^{-1}. a Temperature-dependent fluorescence spectra of a 1:1.5 mixture of **Ant** and **Nap** ([**Ant**] = 1.0 × 10^{-5} M; [**Nap**] = 1.5 × 10^{-5} M) upon cooling the MCH solution from 373 to 293 K. The excitation wavelength was 330 nm. **b** Plot of the fluorescence intensity change (Δ*FL*) for **Ant** at 426 nm (blue) and **Nap** at 557 nm (red) as a function of the temperature upon cooling pure MCH solutions (open circles and squares with dashed lines) and a 1:1.5 mixture (closed circles and squares with solid lines).

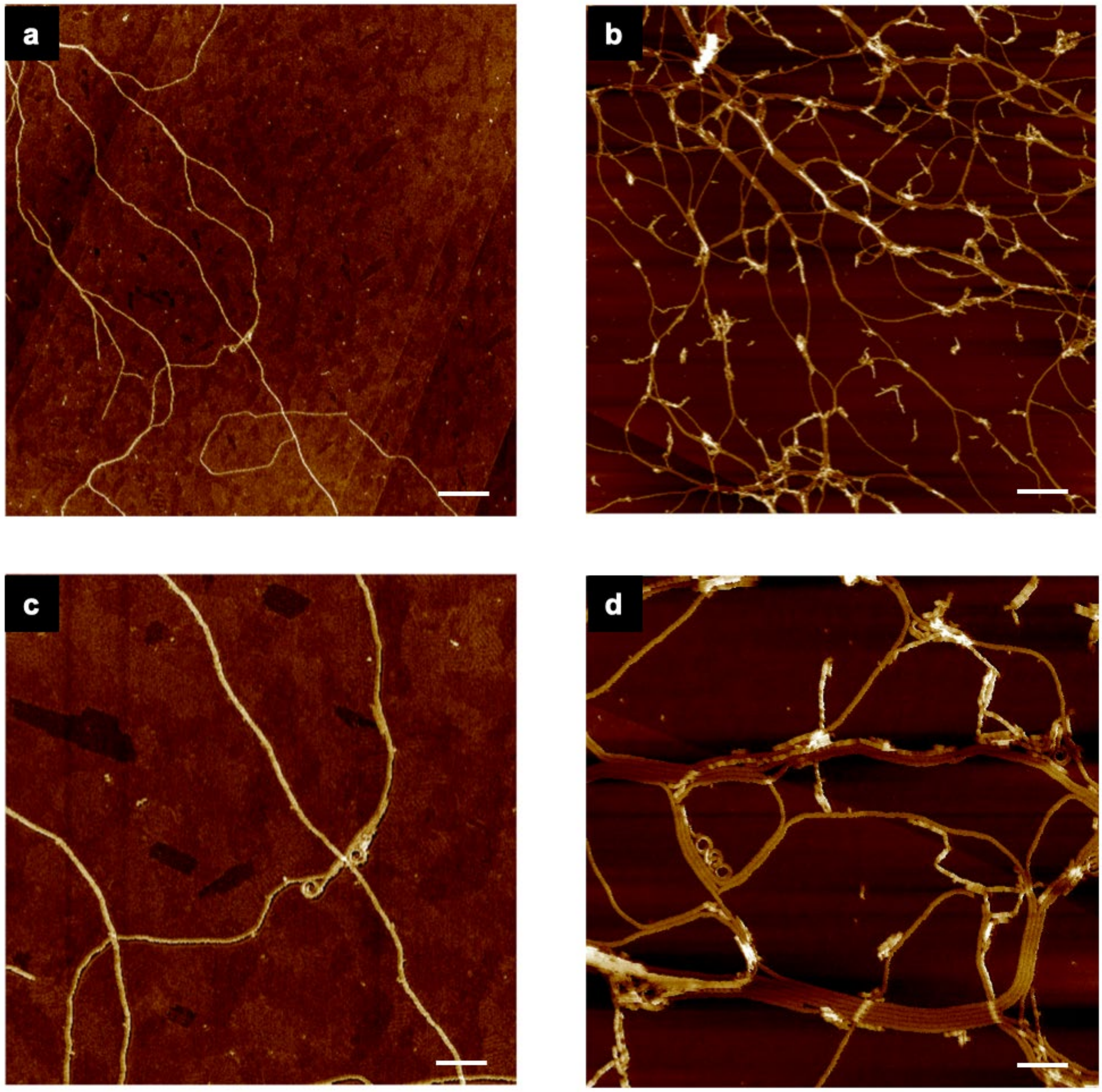

Supplementary Figure 12. AFM images of supramolecular copolymers prepared from the 1:0.5 mixture of Ant and Nap. Additional AFM images of the supramolecular polymers formed upon cooling a hot MCH solution of the 1:0.5 mixture of **Ant** and **Nap** ([**Ant**] = 1.0 × 10^{-5} M; [**Nap**] = 0.5 × 10^{-5} M) from 373 to 293 K at a cooling rate of 1.0 K min^{-1}. Scale bars, 300 nm (**a**,**b**) or 100 nm (**c**,**d**).

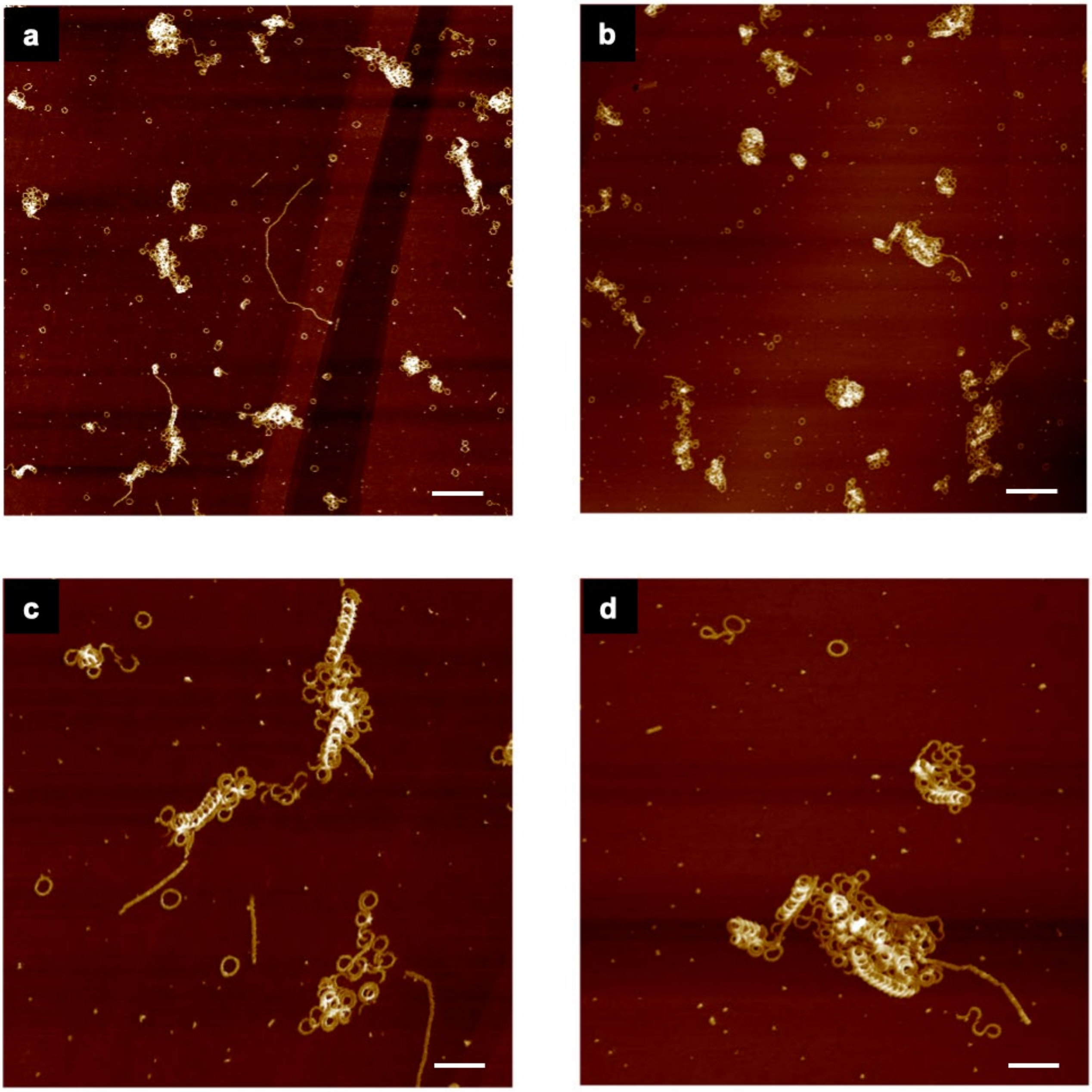

Supplementary Figure 13. AFM images of the supramolecular copolymers prepared from the 1:2 mixture of Ant and Nap. Additional AFM images of the supramolecular copolymers prepared by cooling a hot MCH solution of the 1:2 mixture of **Ant** and **Nap** ([**Ant**] = 1.0×10^{-5} M; [**Nap**] = 2.0×10^{-5} M) from 373 to 293 K at a cooling rate of 1.0 K min^{-1}. Scale bars, 300 nm (**a**,**b**) or 100 nm (**c**,**d**).

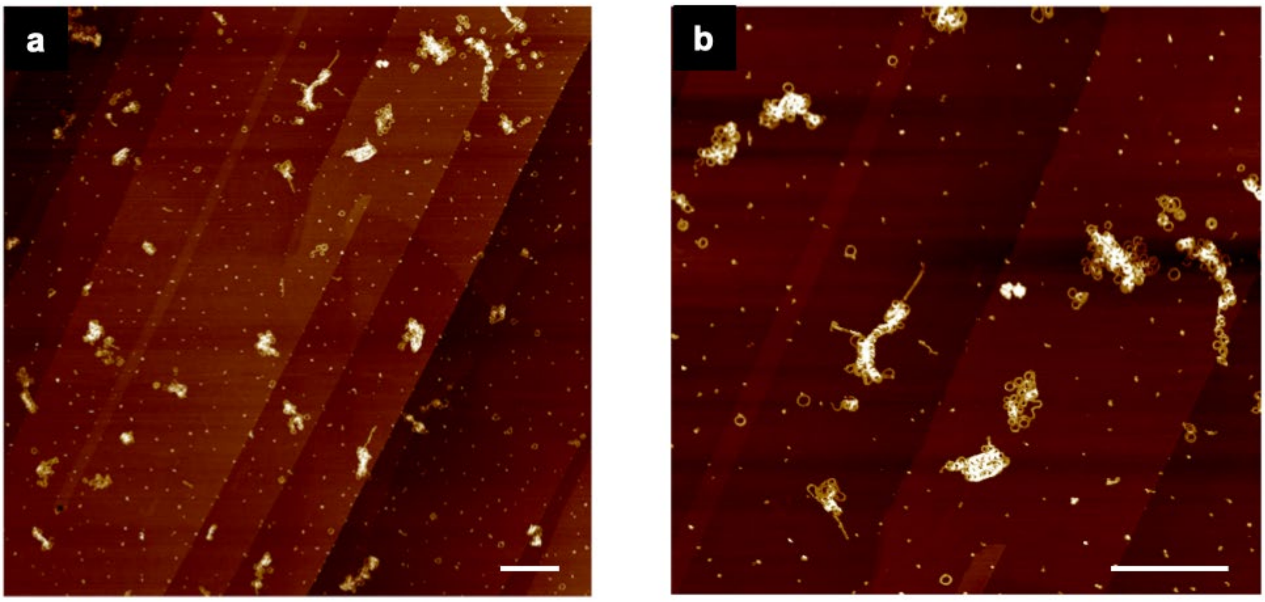

Supplementary Figure 14. AFM images of the supramolecular copolymers prepared from the 1:2.5 mixture of Ant and Nap. Additional AFM images of the supramolecular copolymers prepared by cooling a hot MCH solution of 1:2.5 mixture of **Ant** and **Nap** ([**Ant**] = 1.0 × 10^{-5} M; [**Nap**] = 2.5 × 10^{-5} M) from 373 to 293 K at a cooling rate of 1.0 K min^{-1}. Scale bars, 400 nm (**a**,**b**).

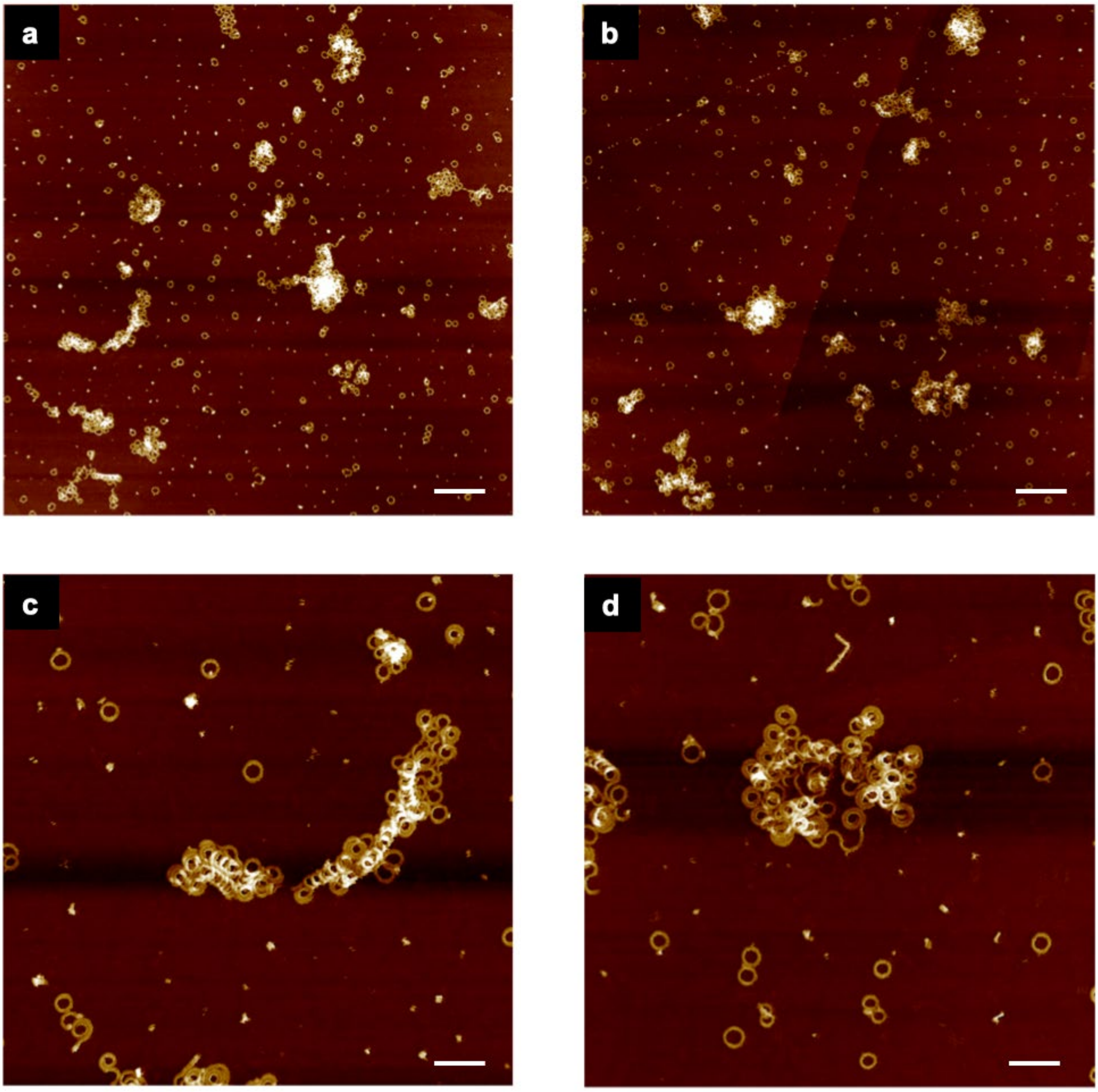

Supplementary Figure 15. AFM images of the supramolecular copolymers prepared from the 1:3 mixture of Ant and Nap. Additional AFM images of the supramolecular copolymers prepared by cooling a hot MCH solution of 1:3 mixture of **Ant** and **Nap** ([**Ant**] = 1.0×10^{-5} M; [**Nap**] = 3.0×10^{-5} M) from 373 to 293 K at a cooling rate of 1.0 K min^{-1}. Scale bars, 300 nm (**a**,**b**) or 100 nm (**c**,**d**).

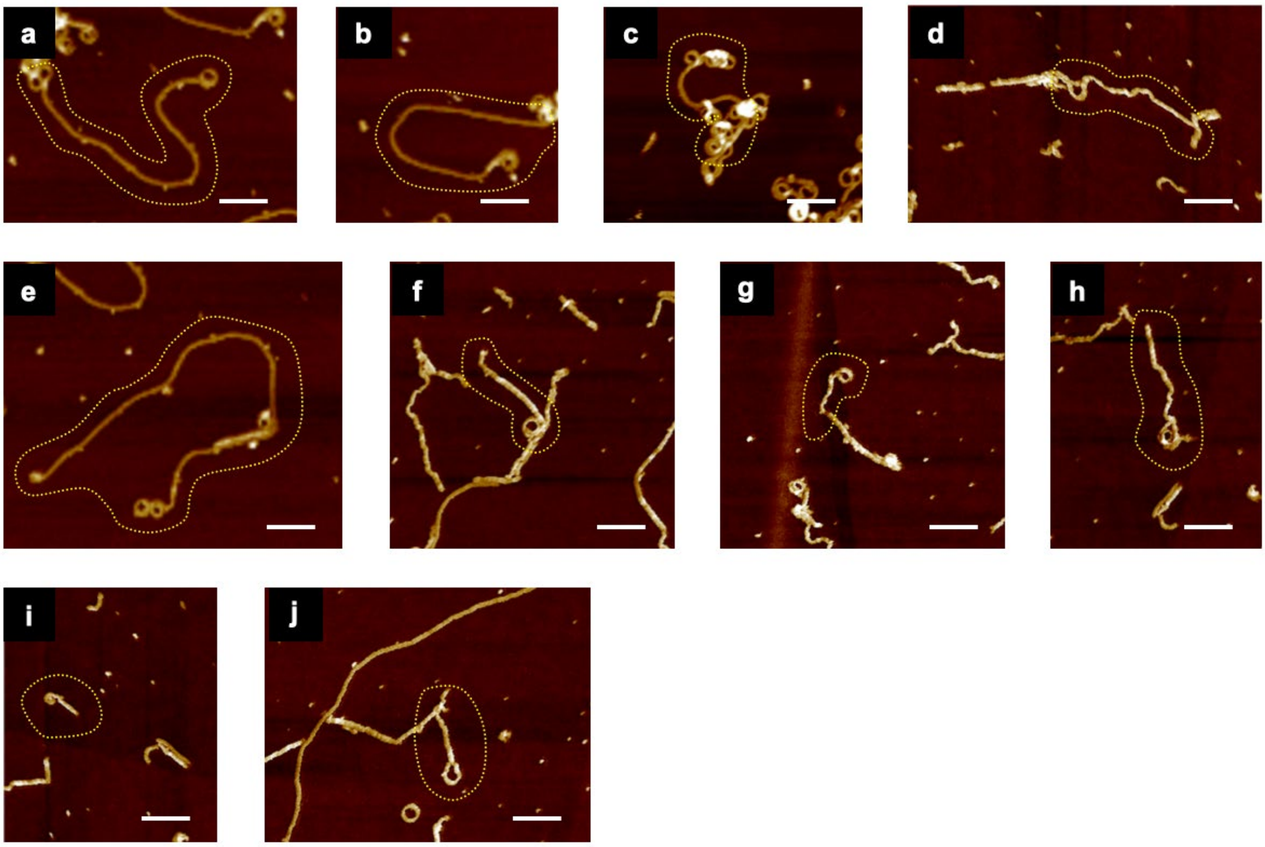

Supplementary Figure 16. AFM images of the supramolecular copolymers prepared from the 1:1 mixture of Ant and Nap. Additional AFM images of the supramolecular copolymers prepared by cooling a hot MCH solution of 1:1 mixture of **Ant** and **Nap** ([**Ant**] = 1.0 × 10^{-5} M; [**Nap**] = 1.0 × 10^{-5} M) from 373 to 293 K at a cooling rate of 1.0 K min^{-1}. Scale bars, 100 nm.

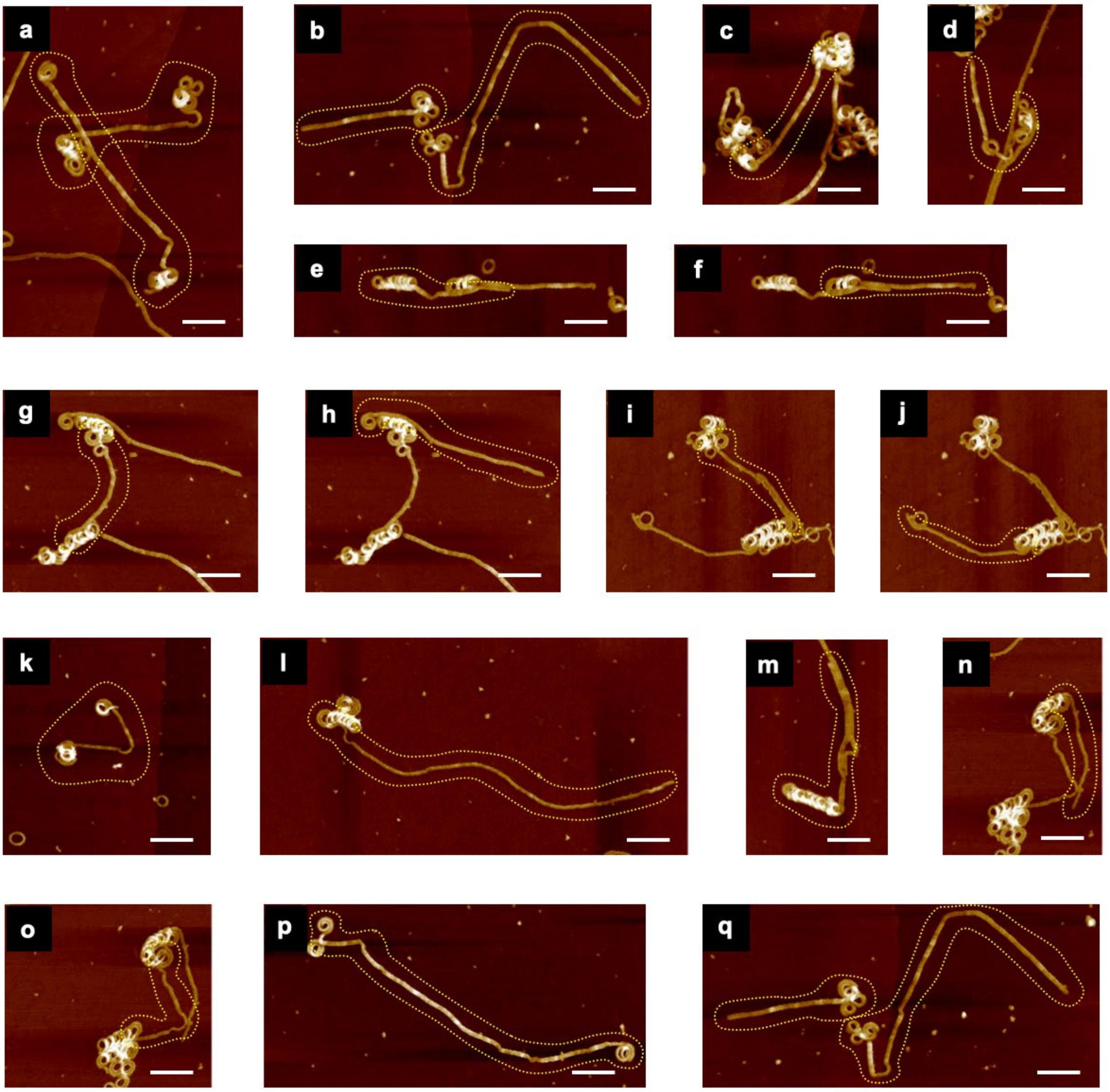

Supplementary Figure 17. AFM images of the supramolecular copolymers prepared from the 1:1.3 mixture of Ant and Nap. Additional AFM images of the supramolecular copolymers prepared by cooling a hot MCH solution of 1:1.3 mixture of **Ant** and **Nap** ([**Ant**] = 1.0 × 10^{-5} M; [**Nap**] = 1.3 × 10^{-5} M) from 373 to 293 K at a cooling rate of 1.0 K min^{-1}. Scale bars, 100 nm.

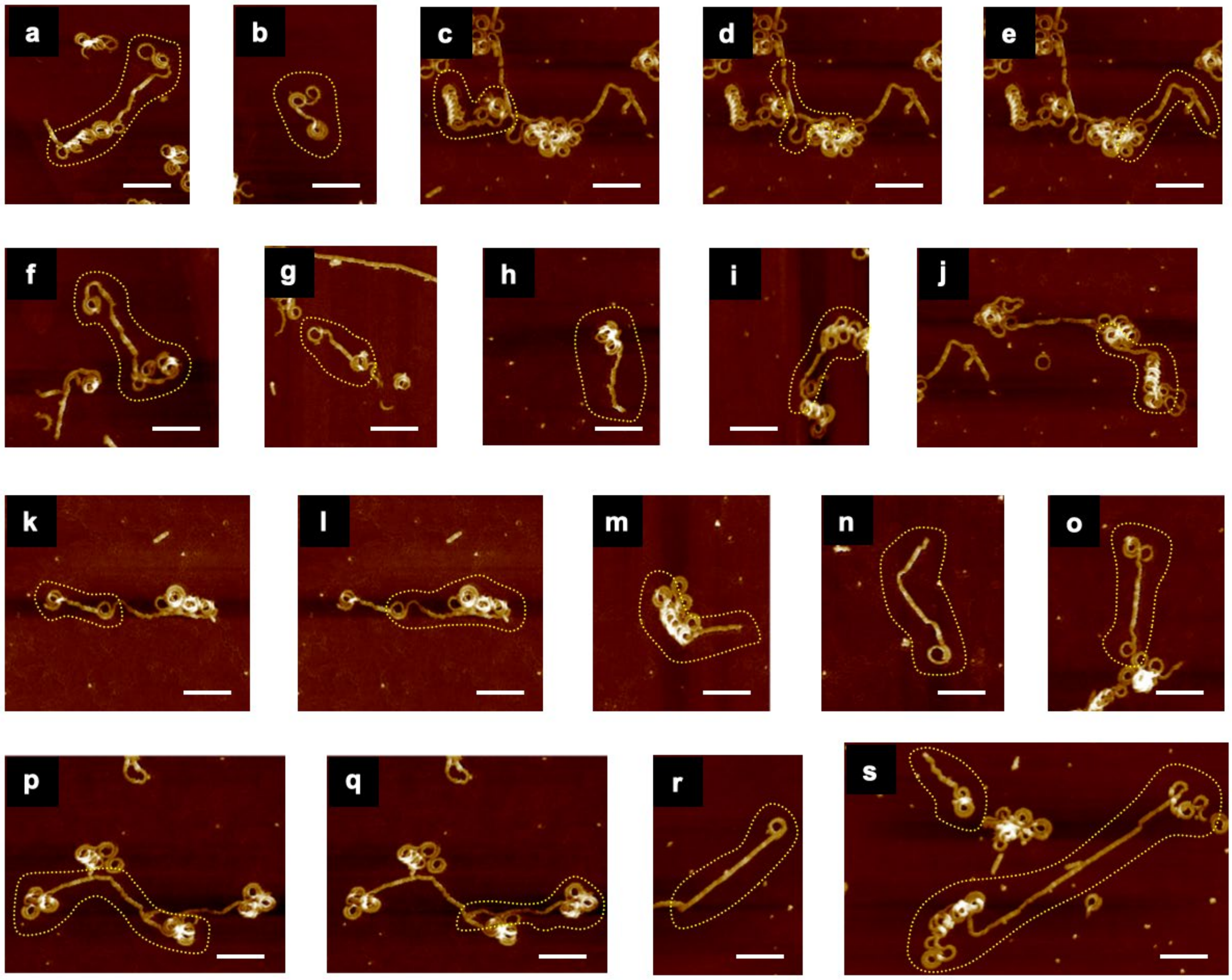

Supplementary Figure 18. AFM images of the supramolecular copolymers prepared from the 1:1.5 mixture of Ant and Nap. Additional AFM images of the supramolecular copolymers prepared by cooling a hot MCH solution of 1:1.5 mixture of **Ant** and **Nap** ([**Ant**] = 1.0 × 10^{-5} M; [**Nap**] = 1.5 × 10^{-5} M) from 373 to 293 K at a cooling rate of 1.0 K min^{-1}. Scale bars, 100 nm.

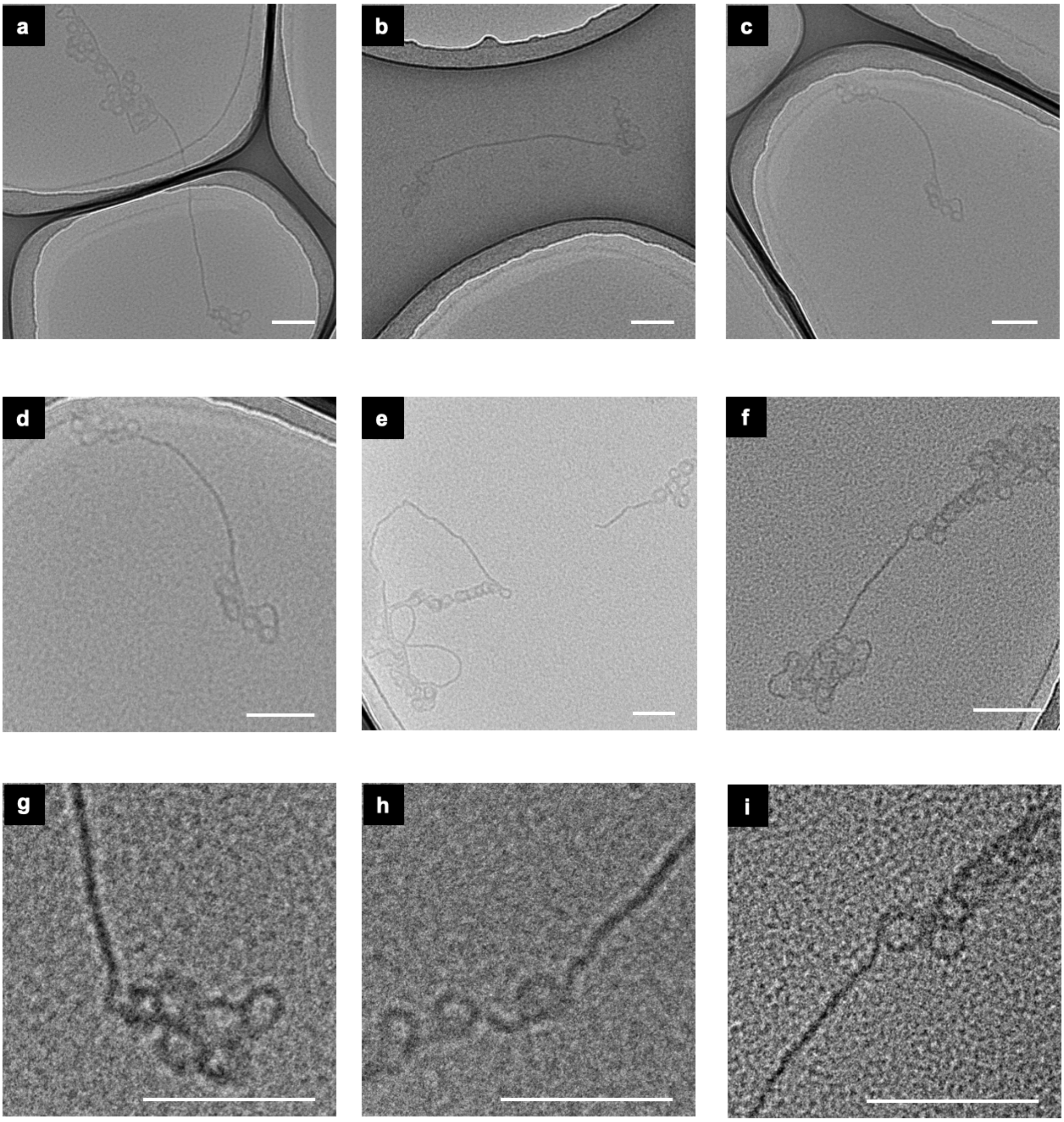

Supplementary Figure 19. TEM images of supramolecular copolymers prepared from the 1:1.5 mixture of Ant and Nap. TEM images of the supramolecular copolymers prepared by cooling a hot MCH solution of 1:1.5 mixture of **Ant** and **Nap** ([**Ant**] = 1.0×10^{-5} M; [**Nap**] = 1.5×10^{-5} M) from 373 to 293 K at a cooling rate of 1.0 K min^{-1}. Scale bars, 100 nm.

Supplementary Table 1. Degree of polymerization (*DP*) of chimeric fibers and its linear and coiled segments evaluated with the AFM analysis.

	Chimeric fibers	Linear segments in chimeric fibers (DP_{linear})	Coiled segments in chimeric fiber (DP_{coil})
1:1.3 mixture	2.1×10^3 ($l_{av} = 7.0 \times 10^2$ nm)[a]	1.1×10^3 ($l_{av} = 4.3 \times 10^2$ nm)[a]	9.7×10^2 ($l_{av} = 2.7 \times 10^2$ nm)[a]
1:1.5 mixture	1.6×10^3 ($l_{av} = 4.9 \times 10^2$ nm)[a]	5.5×10^2 ($l_{av} = 2.0 \times 10^2$ nm)[a]	1.0×10^3 ($l_{av} = 2.8 \times 10^2$ nm)[a]

[a] l_{av}: average length of chimeric fibers and each segment in the chimeric fibers.

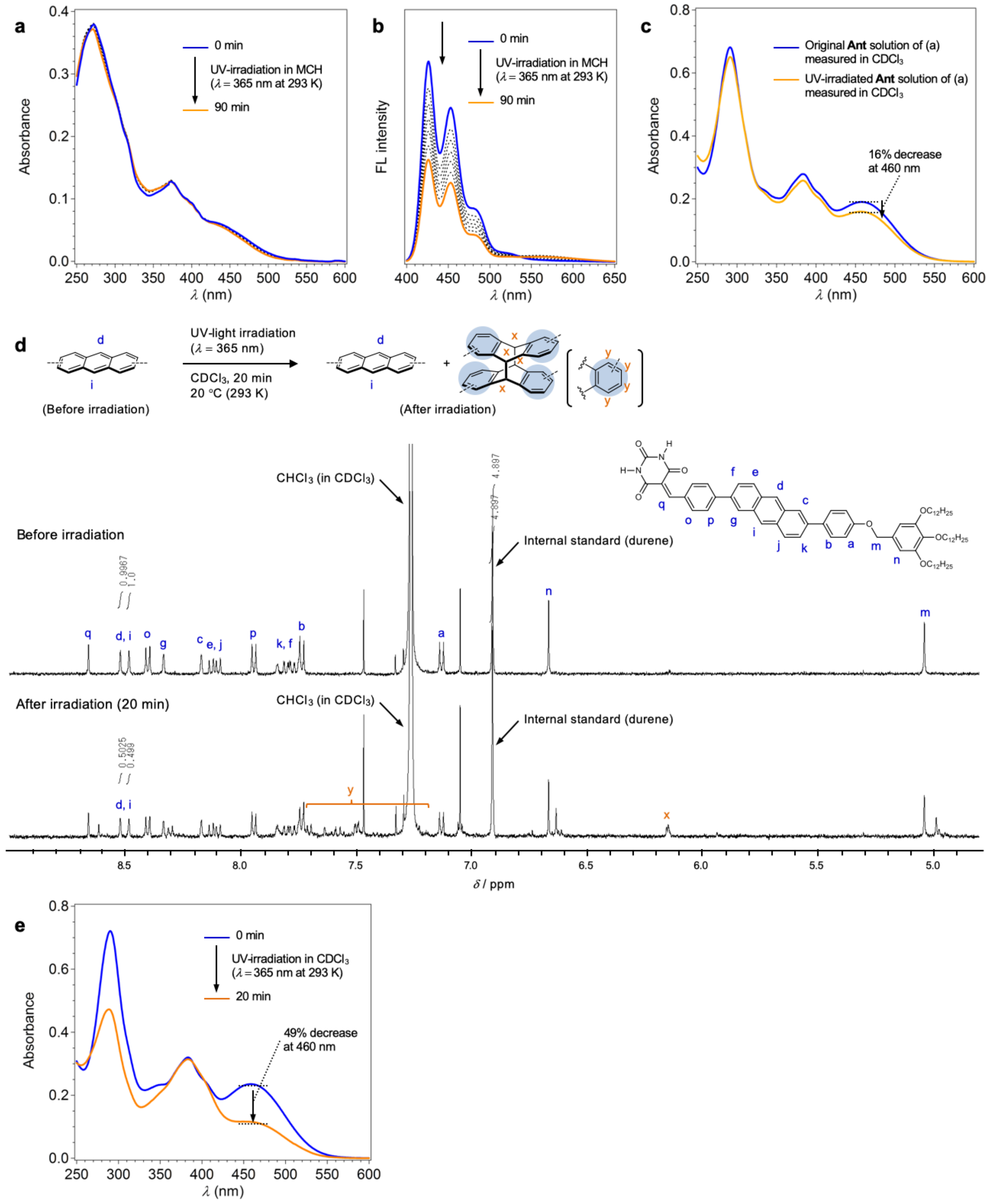

Supplementary Fig. 20. UV-vis, fluorescence and NMR studies on UV-irradiation of Ant supramolecular polymers. a,**b** UV-vis absorption (**a**) and fluorescence spectral changes (**b**, λ_{ex} = 385 nm) of **Ant** ($c = 1.0 \times 10^{-5}$ M) in MCH upon irradiation with UV light (λ = 365 nm) for 90 min at 293 K. The solution was prepared by cooling a hot MCH solution from 373 to 293 K at a cooling rate of 1.0 K min^{-1}. **c** The UV-vis absorption spectra of original and UV-irradiated **Ant** solution shown in (**a**) after solvent replacement of MCH with $CDCl_3$. The photo-conversion after 90-min

irradiation was estimated to be 16% by ^{1}H NMR and UV-vis absorption analyses shown in (**d**,**e**). **d** ^{1}H NMR spectra of **Ant** (c = 1.0 × 10^{-4} M) in $CDCl_3$ before and after irradiation with UV light for 20 min at 293 K. 1,2,4,5-Tetramethylbenzenedurene (durene, 2.45 equiv.) was used as an internal standard. From the integration of the signals *d* and *i* assignable to anthracene aromatic protons, the photo-conversion is estimated to be 50%. **e** UV-vis spectra of the $CDCl_3$ solutions (c = 1.0 × 10^{-5} M) prepared by diluting the solutions used for the above NMR measurement. The absorption intensity at 460 nm, mainly contributed by the anthracene moiety, showed 49% decreased upon the UV-irradiation. This decrease corresponds to the photo-conversion of 50% as estimated by the NMR measurement. This relationship between the absorption and the NMR spectra was used as a reference to estimate the photo-conversion in MCH.

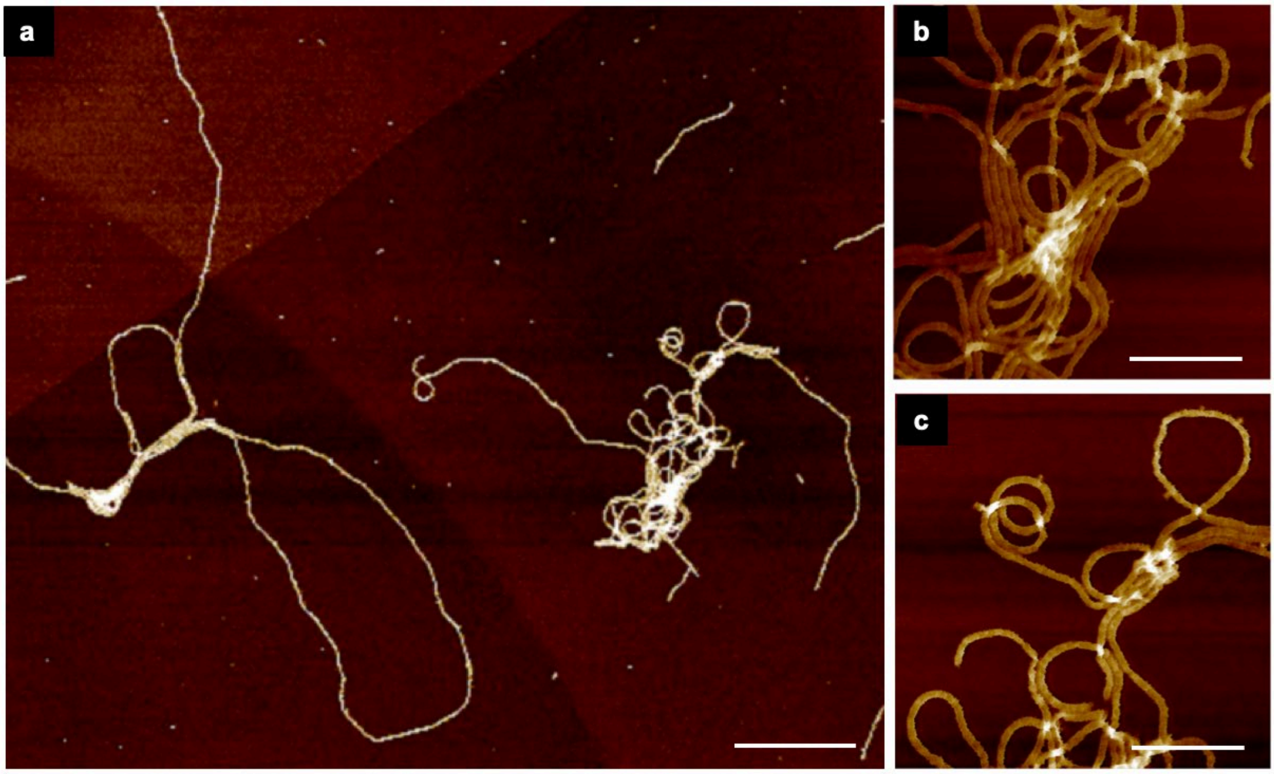

Supplementary Figure 21. UV-induced morphology change of Ant supramolecular polymers. Additional AFM images of supramolecular polymers of **Ant** that were inhomogeneously curved by irradiation with UV light (λ = 365 nm) at 293 K for 90 min. The original linear fiber was prepared by cooling a hot MCH solution of **Ant** (c = 1.0 × 10^{-5} M) from 373 to 293 K at a cooling rate of 1.0 K min^{-1}. Scale bars, 300 nm (**a**) or 100 nm (**b**,**c**).

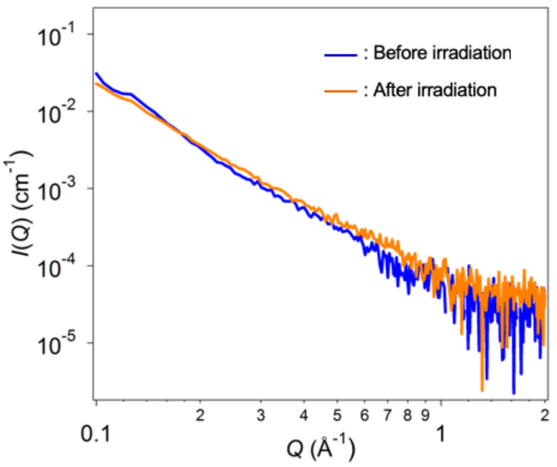

Supplementary Figure 22. SAXS profiles of Ant supramolecular polymers before and after UV-irradiation. SAXS profiles of MCH solutions of **Ant** (c = 2.0 × 10^{-5} M) prepared by cooling a hot MCH solution from 373 to 293 K at a cooling rate of 1.0 K min^{-1} before (blue curve) and after irradiation (orange curve) with UV light (λ = 365 nm) at 293 K for 90 min.

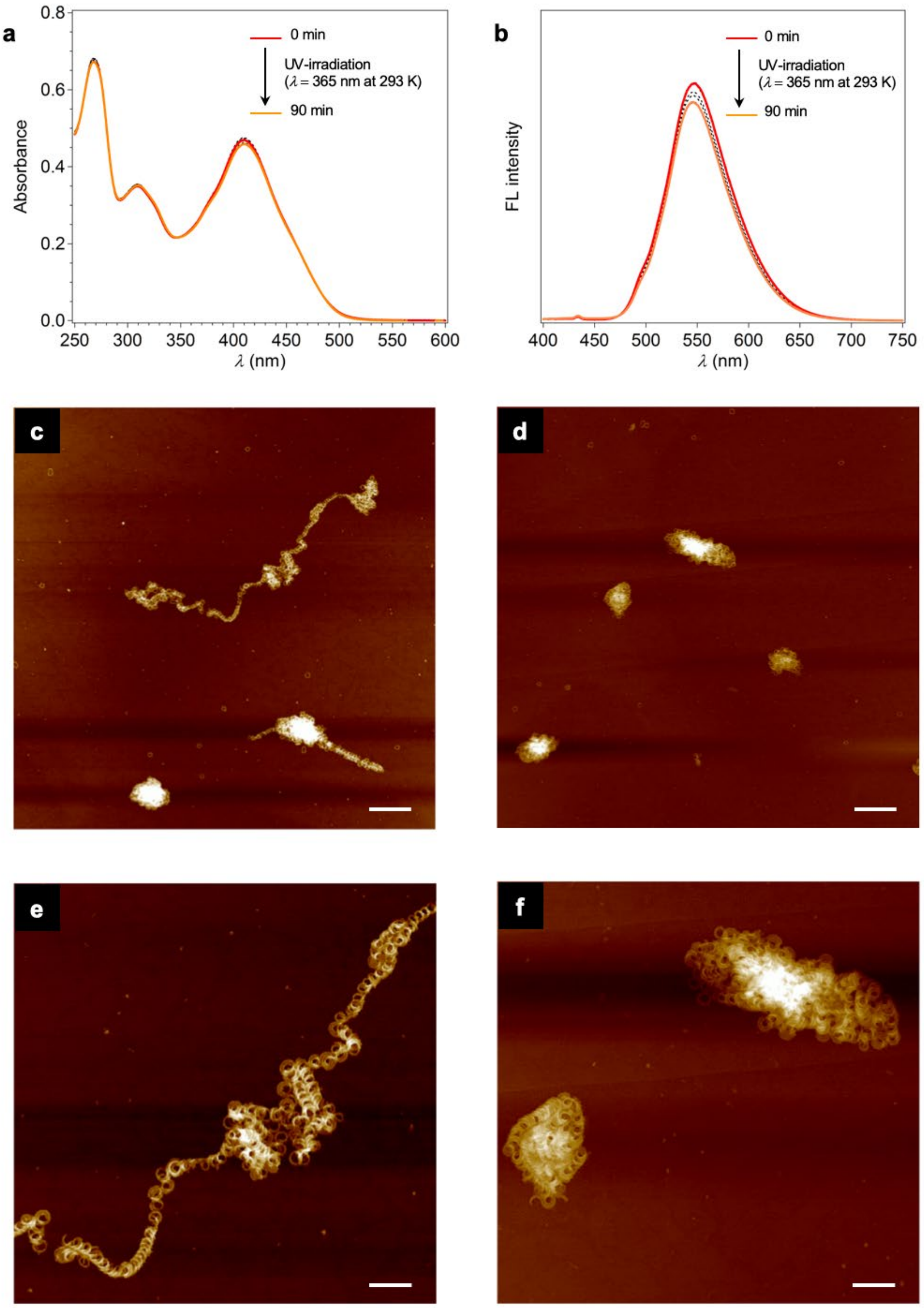

Supplementary Figure 23. UV-vis, fluorescence and AFM studies on UV-irradiation of Nap supramolecular polymers. a,**b** UV-vis absorption (**a**) and fluorescence spectral changes (**b**, λ_{ex} = 385 nm) of **Nap** upon irradiation with UV light (λ = 365 nm,) for 90 min at 293 K. The solution was prepared by cooling a hot MCH solution of **Nap** (c = 1.5 × 10^{-5} M) from 373 to 293 K at a cooling rate of 1.0 K min^{-1}. **c**–**f** AFM images of supramolecular polymers of **Nap** upon irradiation with UV light (λ = 365 nm) at 293 K for 90 min. Scale bars, 300 nm (**c**,**d**) or 100 nm (**e**,**f**).

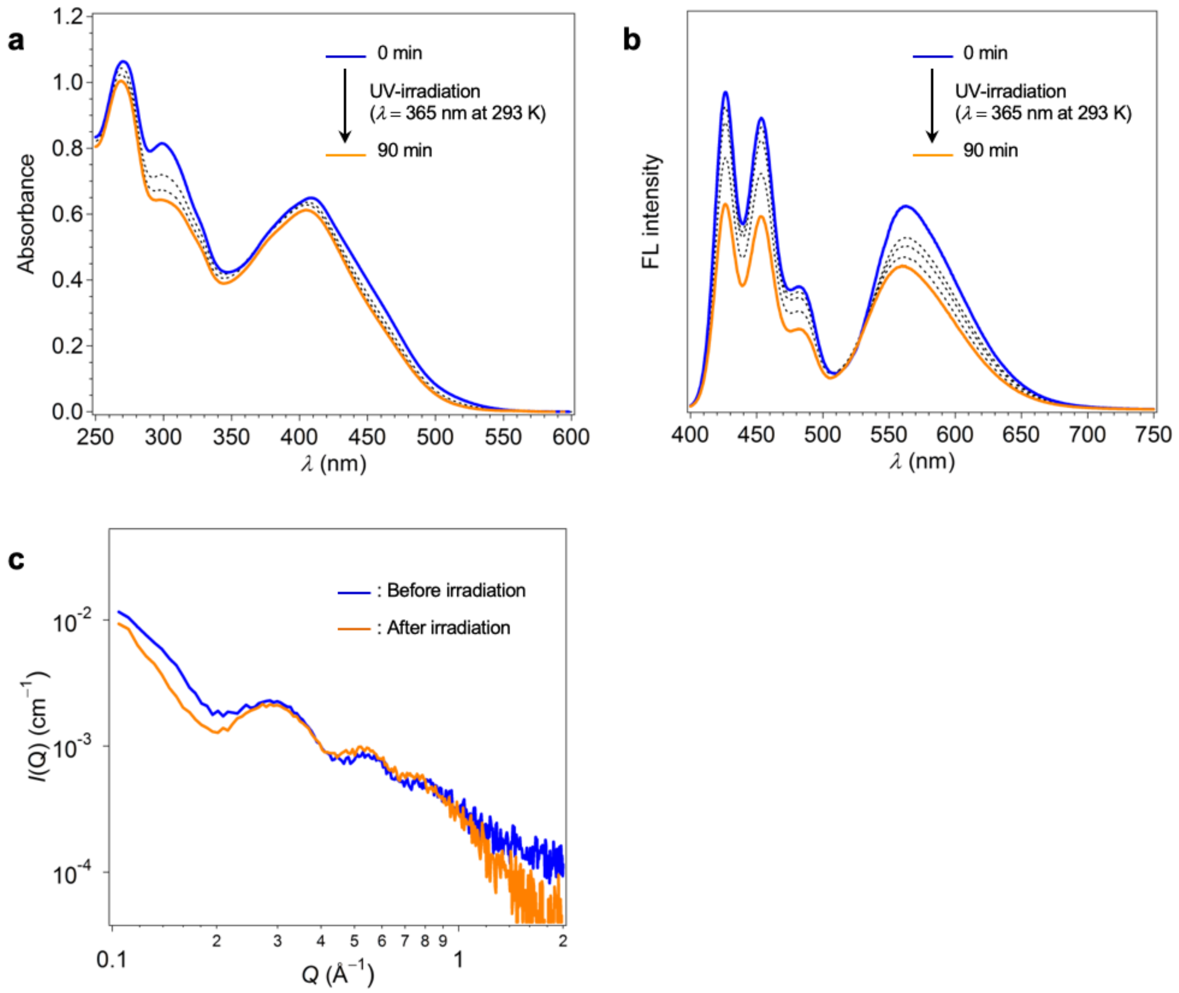

Supplementary Figure 24. UV-vis, fluorescence and SAXS studies on UV-irradiation of chimeric fibers with linear–coiled topology. a,**b** UV-vis absorption (**a**) and fluorescence spectral changes (**b**, λ_{ex} = 385 nm) of chimeric fibers upon irradiation with UV light (λ = 365 nm) for 90 min at 293 K. The solution was prepared by cooling a hot MCH solution of the 1:1.5 mixture of **Ant** and **Nap** ([**Ant**] = 1.0×10^{-5} M; [**Nap**] = 1.5×10^{-5} M) from 373 to 293 K at a cooling rate of 1.0 K min^{-1}. **c** SAXS profiles of MCH solutions of the 1:1.5 mixture of **Ant** and **Nap** before (blue curve) and after irradiation (orange curve) with UV light (λ = 365 nm) at 293 K for 90 min. The original solution was prepared by cooling a hot MCH solution of **Ant** and **Nap** ([**Ant**] = 1.0×10^{-5} M; [**Nap**] = 1.5×10^{-5} M) from 373 to 293 K at a cooling rate of 1.0 K min^{-1}. Because the scattering intensity of the original solution was too low to obtain reliable SAXS profiles, the solutions before and after UV-irradiation were concentrated by N_2 flow to increase the concentration nearly double (i.e., [**Ant**] = 2.0×10^{-5} M; [**Nap**] = 3.0×10^{-5} M).

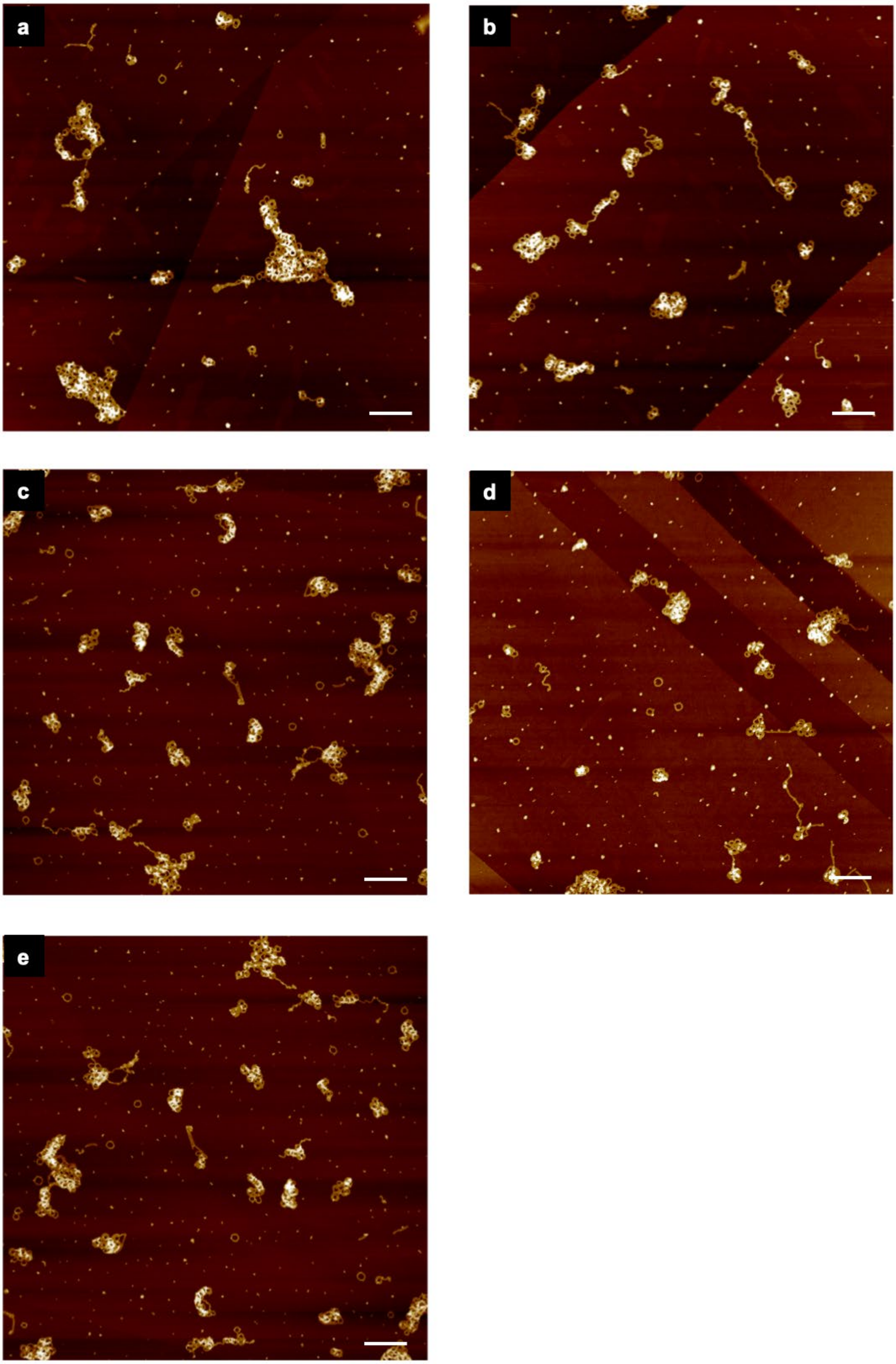
a
b
c
d
e

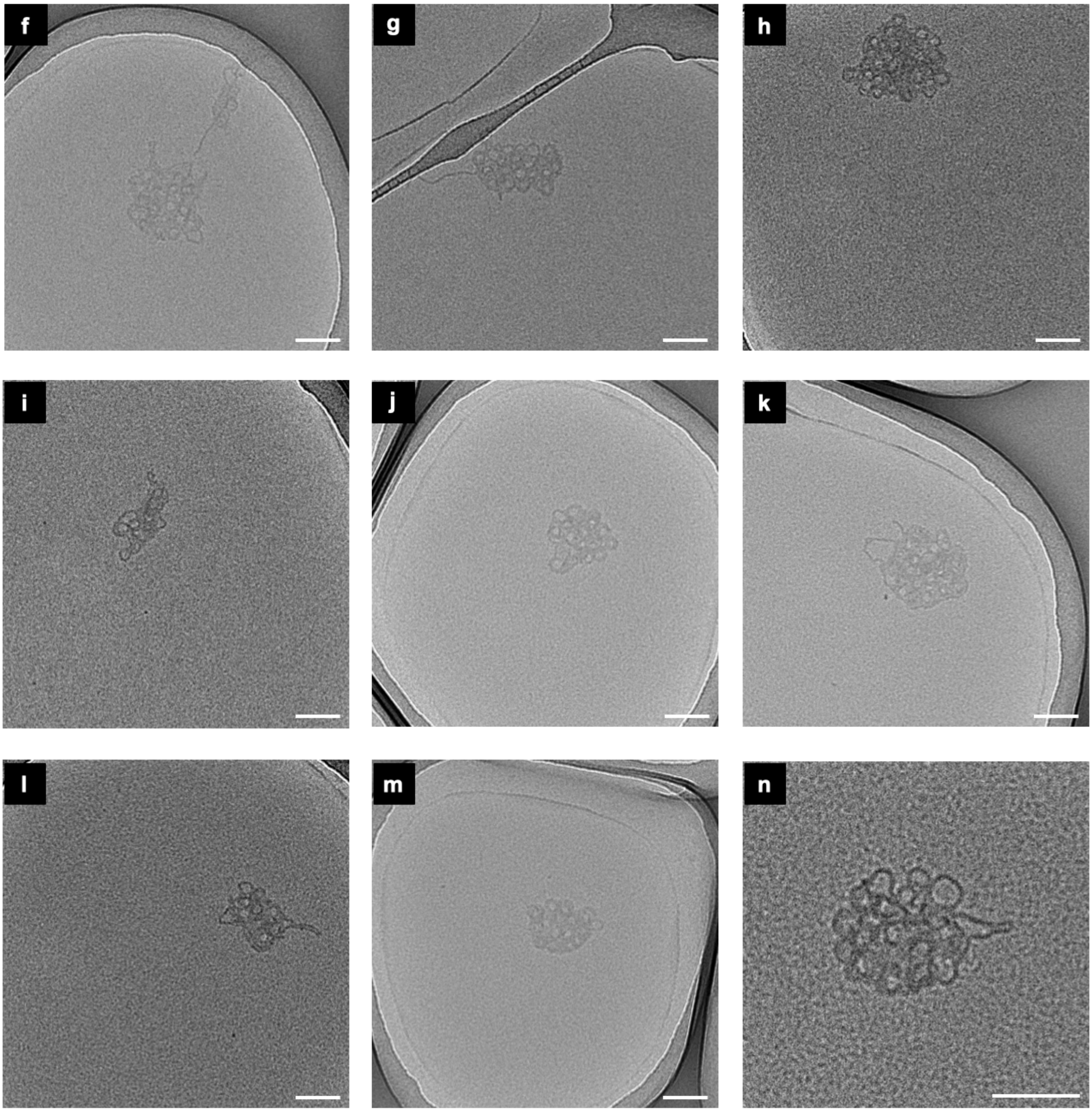

Supplementary Figure 25. AFM and TEM images of UV-irradiated chimeric fibers. a–e Additional AFM images of chimeric fibers folded by irradiation with UV light (λ = 365 nm) at 293 K for 75 (**a**,**b**) and 90 min (**c**–**e**). The original chimeric fibers were prepared by cooling a hot MCH solution of 1:1.5 mixture of **Ant** and **Nap** ([**Ant**] = 1.0 × 10^{-5} M; [**Nap**] = 1.5 × 10^{-5} M) from 373 to 293 K at a cooling rate of 1.0 K min^{-1}. Scale bars, 200 nm. **f**–**n** TEM images of folded chimeric fibers obtained by 90-min UV-irradiation. Scale bars, 100 nm.

a

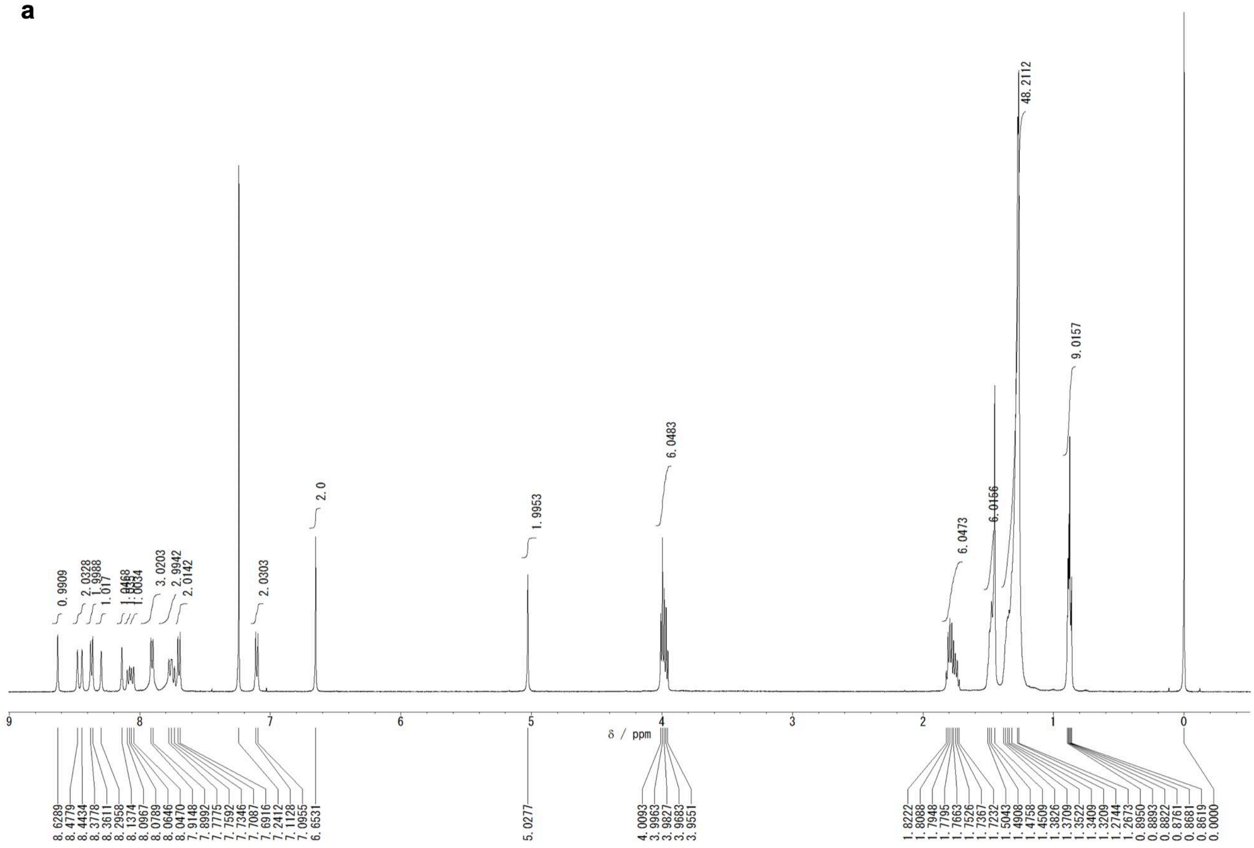

b

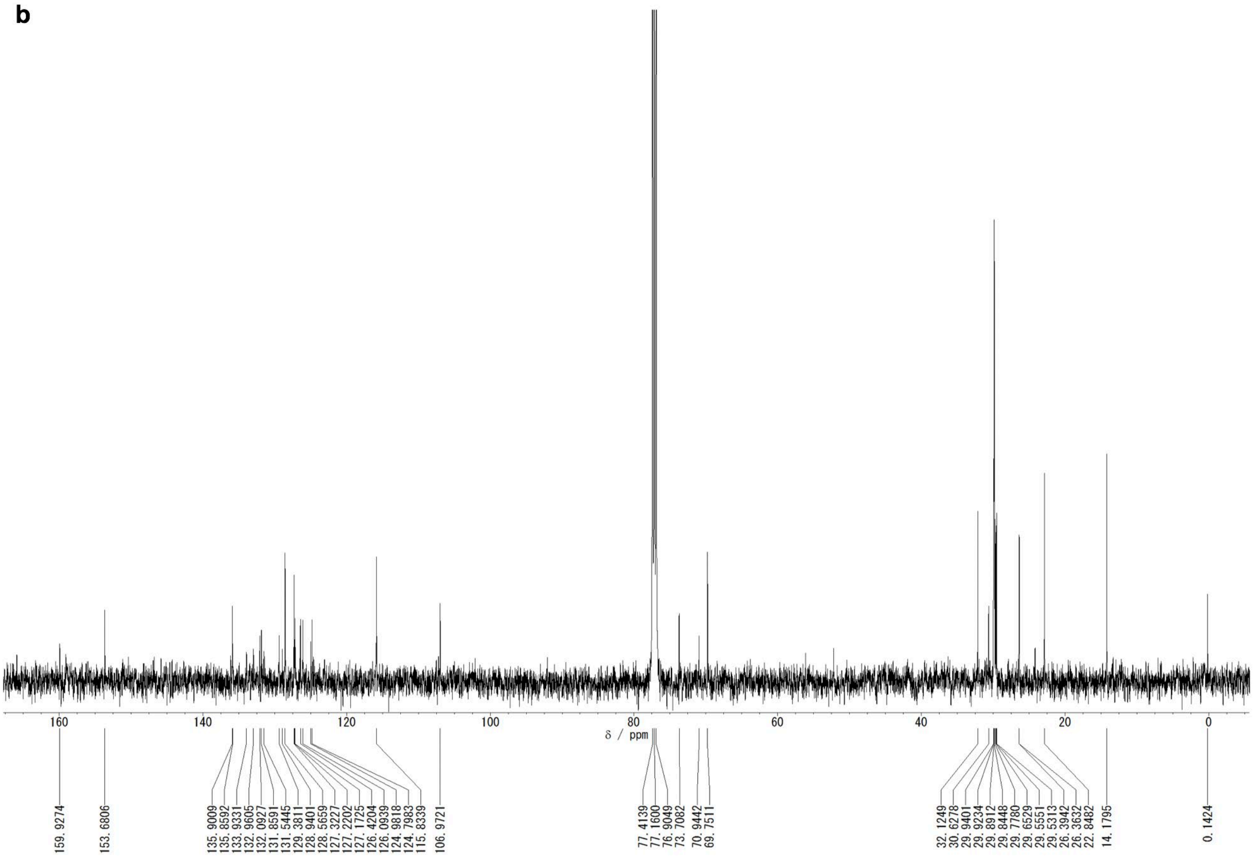

Supplementary Figure 26. ^{1}H NMR and ^{13}C NMR of Ant. ^{1}H NMR (**a**) and ^{13}C NMR (**b**) of **Ant** in $CDCl_3$ at 333 K.

Supplementary References

1. Igarashi, N., Watanabe, Y., Shinohara, Y., Inoko, Y., Matsuba, G., Okuda, H., Mori, T. & Ito, K. Upgrade of the small angle x-ray scattering beamlines at the photon factory. *J. Phys. Conf. Ser.* **272**, 012026 (2011).
2. Shimizu, N., Yatabe, K., Nagatani, Y., Saijyo, S., Kosuge, T. & Igarashi, N. Software development for analysis of small-angle x-ray scattering data. *AIP Conf. Proc.* **1741**, 050017 (2016).
3. Hollamby, M. J., Aratsu, K., Pauw, B. R., Rogers, S. E., Smith, A. J., Yamauchi, M., Lin, X. & Yagai, S. Simultaneous SAXS and SANS analysis for the detection of toroidal supramolecular polymers composed of noncovalent supermacrocycles in solution. *Angew. Chem. Int. Ed.* **55**, 9890–9893 (2016).
4. Balagurusamy, V. S. K., Ungar, G., Percec, V. & Johansson, G. Rational design of the first spherical supramolecular dendrimers self-organized in a novel thermotropic cubic liquid-crystalline phase and the determination of their shape by x-ray analysis. *J. Am. Chem. Soc.* **119**, 1539–1555 (1997).
5. Yagai, S., Suzuki, M., Lin, X., Gushiken, M., Noguchi, T., Karatsu, T., Kitamura, A., Saeki, A., Seki, S., Kikkawa, Y., Tani, Y. & Nakayama, K.-i. Dynamic self-correcting nucleophilic aromatic substitution. *Chem. Eur. J.* **20**, 16128–16137 (2014).
6. Lin, X., Suzuki, M., Gushiken, M., Yamauchi, M., Karatsu, T., Kizaki, T., Tani, Y., Nakayama, K.-i., Suzuki, M., Yamada, H., Kajitani, T., Fukushima, T., Kikkawa, Y. & Yagai, S. High-fidelity self-assembly pathways for hydrogen-bonding molecular semiconductors. *Sci. Rep.* **7**, 43098 (2017).
7. Prabhu, D. D., Aratsu, K., Kitamoto, Y., Ouchi, H., Ohba, T., Hollamby, M. J., Shimizu, N., Takagi, H., Haruki, R., Adachi, S.-i. & Yagai, S. Self-folding of supramolecular polymers into bioinspired topology. *Sci. Adv.* **4**, eaat8466 (2018).
8. Jonkheijm, P., van der Schoot, P., Schenning, A. P. H. J. & Meijer, E. W. *Science* **313**, 80–83 (2006).
9. Frisch, M. J. *et al. Gaussian 16* (*Revision B.01*) (Gaussian, Inc., 2016).
